# Monoamine oxidase A and organic cation transporter 3 coordinate intracellular β_1_AR signaling to calibrate cardiac contractile function

**DOI:** 10.1007/s00395-022-00944-5

**Published:** 2022-07-17

**Authors:** Ying Wang, Meimi Zhao, Bing Xu, Sherif M. F. Bahriz, Chaoqun Zhu, Aleksandra Jovanovic, Haibo Ni, Ariel Jacobi, Nina Kaludercic, Fabio Di Lisa, Johannes W. Hell, Jean C. Shih, Nazareno Paolocci, Yang K. Xiang

**Affiliations:** 1 Department of Pharmacology, University of California at Davis, Davis, CA 95616 USA; 2 Department of Pharmaceutical Toxicology, China Medical University, Shenyang, 110122 China; 3 VA Northern California Health Care System, Mather, CA USA; 4 Neuroscience Institute, National Research Council of Italy, Padua, Italy; 5 Institute for Pediatric Research Città Della Speranza, Padua, Italy; 6 Department of Biomedical Sciences, University of Padova, Padua, Italy; 7 Department of Pharmacology and Pharmaceutical Sciences, University of Southern California, Los Angeles, CA USA; 8 Division of Cardiology, Johns Hopkins Medical Institutions, Baltimore, MD USA

**Keywords:** β_1_ Adrenergic receptor, Monoamine oxidase A, Organic cation transporter 3, Excitation–contraction coupling, Sarcoplasmic reticulum, Cardiac contraction

## Abstract

We have recently identified a pool of intracellular β_1_ adrenergic receptors (β_1_ARs) at the sarcoplasmic reticulum (SR) crucial for cardiac function. Here, we aim to characterize the integrative control of intracellular catecholamine for subcellular β_1_AR signaling and cardiac function. Using anchored Förster resonance energy transfer (FRET) biosensors and transgenic mice, we determined the regulation of compartmentalized β_1_AR-PKA signaling at the SR and plasma membrane (PM) microdomains by organic cation transporter 3 (OCT3) and monoamine oxidase A (MAO-A), two critical modulators of catecholamine uptake and homeostasis. Additionally, we examined local PKA substrate phosphorylation and excitation–contraction coupling in cardiomyocyte. Cardiac-specific deletion of MAO-A (MAO-A-CKO) elevates catecholamines and cAMP levels in the myocardium, baseline cardiac function, and adrenergic responses. Both MAO-A deletion and inhibitor (MAOi) selectively enhance the local β_1_AR-PKA activity at the SR but not PM, and augment phosphorylation of phospholamban, Ca^2+^ cycling, and myocyte contractile response. Overexpression of MAO-A suppresses the SR-β_1_AR-PKA activity and PKA phosphorylation. However, deletion or inhibition of OCT3 by corticosterone prevents the effects induced by MAOi and MAO-A deletion in cardiomyocytes. Deletion or inhibition of OCT3 also negates the effects of MAOi and MAO-A deficiency in cardiac function and adrenergic responses in vivo. Our data show that MAO-A and OCT3 act in concert to fine-tune the intracellular SR-β_1_AR-PKA signaling and cardiac fight-or-flight response. We reveal a drug contraindication between anti-inflammatory corticosterone and anti-depressant MAOi in modulating adrenergic regulation in the heart, providing novel perspectives of these drugs with cardiac implications.

**Supplementary Information:**

The online version contains supplementary material available at 10.1007/s00395-022-00944-5.

## Introduction

During fight-or-flight responses, activation of the sympathetic nervous system releases catecholamines to stimulate βARs in the heart, enhancing heart rate, cardiac contractility, and cardiac output [30]. Blunted βAR and cardiac responses to catecholamines hallmark cardiac dysfunction in heart failure (HF) [2]. Restoring the βAR responsiveness represents a critical therapeutic strategy to rescue cardiac function. Our previous studies suggest targeting monoamine oxidase A (MAO-A) and organic transporter 3 (OCT3) as promising strategies for modulating the intracellular β_1_AR signaling in cardiomyocytes [57, 58]. However, a functional crosstalk of targeting cardiac MAO-A and OCT3 remains unknown.

In addition to the plasma membrane, G protein-coupled receptors (GPCRs) are present and function at intracellular membranes, including endosomes, the Golgi, the sarcoplasmic reticulum (SR), nuclear membranes, and mitochondrial [2, 6, 11, 12, 34, 40, 43, 53, 55, 59]. We have recently identified a pool of β_1_ARs residing on the SR (SR-β_1_ARs), which associate with SR Ca^2+^ ATPase 2a (SERCA2a) but not ryanodine receptor 2 (RyR2). Stimulation of SR-β_1_ARs promotes local protein kinase A (PKA) activity for phosphorylation of phospholamban (PLB) and enhances Ca^2+^ cycling in excitation–contraction (E–C) coupling [57, 58]. The intracellular SR-β_1_ARs produce spatially biased signaling and distinct effects from the β_1_ARs at the PM, providing a framework to selectively fine-tune the local βAR signals and develop therapeutic strategies.

Both OCT3 and MAO-A can tune the cytoplasmic catecholamines by modulating catecholamine uptake or degradation, respectively. In a classic paradigm, catecholamines (norepinephrine, NE, and epinephrine, EPI) released from the sympathetic ganglia neurons activate the β_1_ARs on the PM (PM-β_1_ARs) to enhance heart rate, cardiac contractility, and cardiac output [30]. The majority (90%) of catecholamines are reabsorbed back into the cytoplasm in neurons. The myocardium absorbs about 5% of catecholamines through OCT3 [13]. In fact, the catecholamines in myocytes can activate the intracellular adrenergic receptors [36, 57, 61], including the SR-β_1_ARs, which synergize with the PM-β_1_AR signaling to promote E–C coupling [5, 36, 47, 57, 61]. Additionally, MAO-A oxidizes intracellular catecholamines, thereby negatively regulating the SR-β_1_ARs signaling [27, 58]. It is still unknown whether MAO-A and OCT3 functionally interact to coordinate spatial–temporal regulation of subcellular β_1_AR and cardiac fight-or-flight response.

Both OCT3 and MAO-A are associated with HF development, which is featured by desensitized βAR signaling [21, 27, 28, 58]. Increased MAO-A expression and activity correlate with reduced myocardium catecholamine contents, increased reactive oxidative stress (ROS), and impaired β_1_AR function in HF [27, 58]. On the other hand, elevated corticosterone (CORTI), an endogenous OCT3 inhibitor, is an independent risk factor for HF [38]. The actions of CORTI in the heart remain unclear. We have recently reported that activation of the SR-β_1_ARs is governed by the OCT3-mediated catecholamine transport into myocytes. CORTI suppresses the intracellular SR-β_1_AR signaling and inotropic response by inhibiting OCT3. Therefore, we hypothesize that the intracellular catecholamine levels, β_1_AR signaling, and cardiac function are dynamically controlled by MAO-A or OCT3; the drugs targeting these proteins may interact in modulating adrenergic stimulation of cardiac function.

In this study, we combined subcellular targeted biosensors with genetic and pharmacological approaches to investigate the interaction between OCT3 and MAO-A in modulating the intracellular SR-β_1_AR signaling and cardiac function. We offer compelling evidence to support that OCT3 and MAO-A calibrate the intracellular β_1_AR signaling and cardiac E–C coupling under catecholamine stimulation. Our data highlight the essential interaction between two classes of clinically used drugs, anti-inflammation CORTI, and anti-depressant MAOi, in modulating the intracellular β_1_AR signaling and cardiac function.

## Materials and methods

### Surgery and animals

C57BL/6J WT, OCT3 knockout (OCT3-KO), MAO-A flox (MAO-A-FF), MAO-A flox/MHC-cre + (cardiac-specific knockout, MAO-A-CKO), and β_1_AR-KO male and female mice at 2–4 months old were randomized and used in this study. AVMs from 3 to 6 months old rabbits were provided by Dr. Donald Bers’s Laboratory. Detailed descriptions of echocardiography, Ca^2+^ imaging, fluorescence resonance energy transfer (FRET), immunoblot, and myocardial infarction surgery are provided in the Online Supplemental Materials and Methods. All animal procedures were performed following the guidelines of the Institutional Animal Care and Use Committee (IACUC, Protocol 20234 and 20957) at the University of California at Davis and followed the NIH and ARRIVE guidelines. Animals were housed in the UC Davis animal facility with automatically controlled humidity (30–70%), temperature (22 °C), and lightning (12/12-h cycle). The facility husbandry staff was responsible for the feeding, watering, and care of animals. The animal strain information was listed in the Animal Resource Table. Mice were anesthetized with isoflurane (1–2%) in oxygen through a nose cone during echocardiography. Mice were humanely euthanized for tissue harvest and cell isolation under deep isoflurane anesthesia (3–5%). The hearts were quickly excised and snap-frozen in liquid nitrogen and then transferred to a − 80 °C freezer for long-term storage. Alternatively, hearts were rinsed promptly in a chilled buffer for cannulation to isolate adult ventricle myocytes (AVMs).

### Statistical analysis

Pooled data were represented as the mean ± SD. Animals were randomized during the experiments. For the statistical analysis, all pairwise comparison was conducted by compare the mean of each column with the mean of every other column. Only *p* values of the interested comparisons were reported given the space limits. We performed fully blinded analyses with different persons carrying out the experiments and analysis. Representative figures/images reflected the average levels of each experiment. Normality of the data (*n* ≥ 6) was assessed using the Shapiro–Wilk test in GraphPad Prism 9 with significance at alpha = 0.05 (GraphPad Inc., San Diego, CA). If *n* < 6, a non-parametric Mann–Whitey test or Kruskal–Wallis test was performed where appropriate. Comparisons between two groups were performed by a two-tailed unpaired *t* test or paired *t* test. Comparisons between more than two groups were performed by one-way ANOVA or two-way ANOVA followed by Tukey’s post hoc using Prism 9.0 software (GraphPad). Paired t tests compare the means of the same group of mice and does not assume equal variance between two groups. For unpaired *t* tests, the variance is assumed to be equal. Paired two-tailed student *t* test or row-matched (RM) one-way AVONA analysis was performed to compare the mean of different measurements taken from the same animals. All comparisons and pairings were designed in original experimental protocol.

## Results

### Corticosterone prevents MAO-A inhibitor from enhancing cardiac fight-or-flight response

**Fig. 1:**
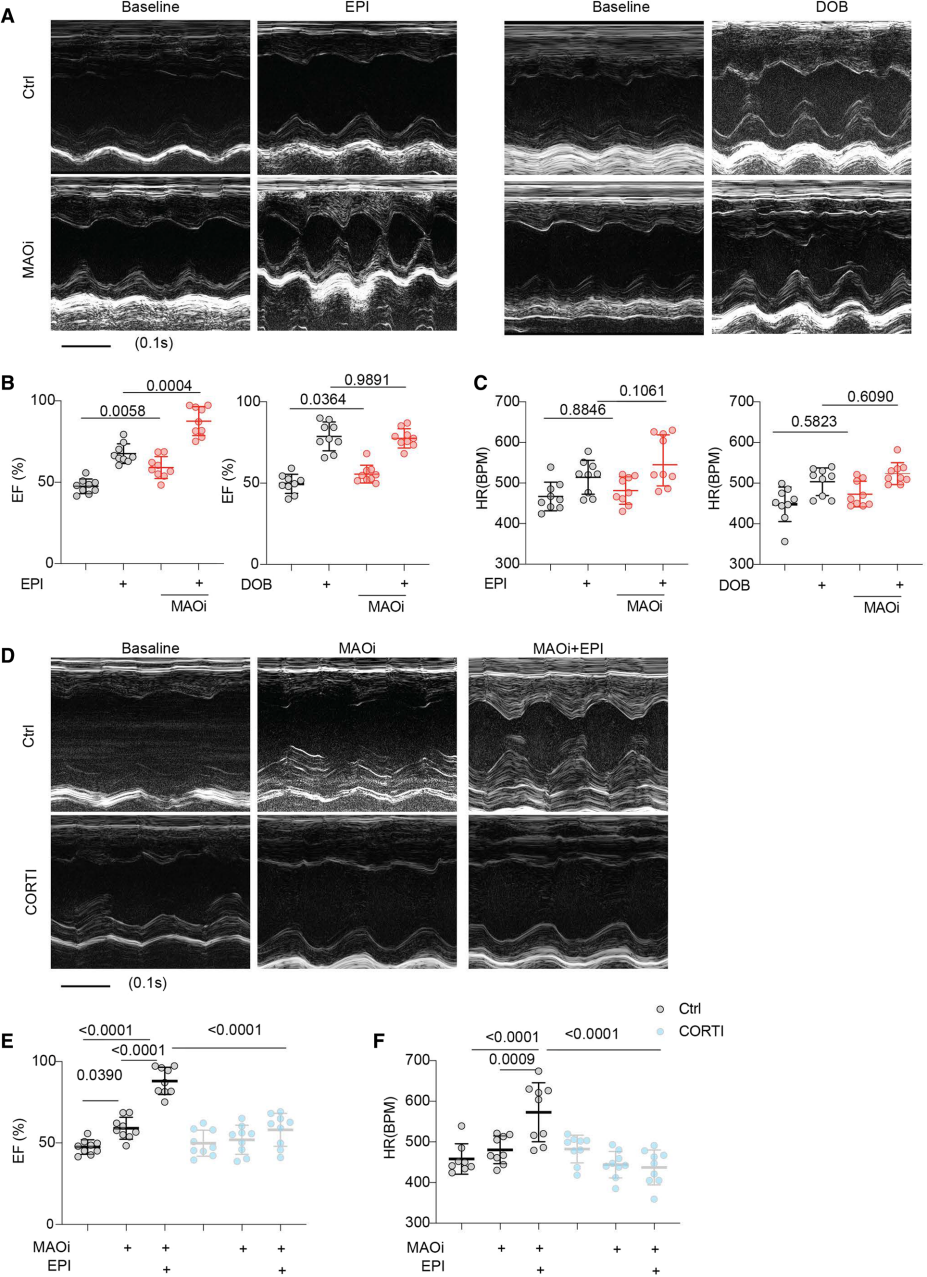
Corticosterone suppresses the MAOi-mediated enhancement of inotropic response in mouse hearts. M-mode echocardiography was recorded for 2 min at baseline and for 8 min after each drug administration. The maximal cardiac response was reported. **A** Representative echocardiography images of WT mouse before and after injection of EPI or DOB (100 µg/kg, *i.p.*) with and without MAOi pretreatment (0.4 mg/kg, *i.p.*, 5 min). **B**, **C** Quantification of mouse cardiac EF and HR as treated in **A**. **D** Representative echocardiography images of WT mouse in response to EPI after pretreatment with MAOi and CORTI (200 μg/kg, *i.p.*, 5 min). **E**, **F** Quantification of maximal EF and HR in **D**. Data are shown as mean ± SD of individual mice. *n* = 9/group, *p* values were obtained by row-matched (RM) one-way ANOVA followed by Tukey’s test

WT mice were challenged with a series of doses of β_1_-agonist dobutamine (DOB, intraperitoneal injection, *i.p.*) to mimic the fight-or-flight response under stress. At 100 μg/kg, DOB produced near-maximal increases in ejection fraction (EF) and heart rate (HR, Fig. 1, Online Figure I, and Online Table 1); and the concentration was chosen for β-agonists in the following in vivo experiments. Our recent study shows that MAOi enhances myocyte shortening by promoting the intracellular SR-β_1_AR signaling induced by catecholamines [58]. Here, treatment with MAOi elevated the baseline EF and enhanced the EPI-induced cardiac EF responses in WT mice (Fig. 1A, B, Online Figure IC, and Online Table 2). As a non-substrate of MAO [63], DOB increased cardiac EF and HR over the baselines, which were not affected by MAOi (Fig. 1A, C, Online Figure ID, and Online Table 3). These data indicate that inhibition of MAO-A enhances the catecholamine-triggered cardiac contractile responses. Interestingly, catecholamines are transported into cardiomyocytes by OCT3 [5, 57, 61]. We assessed whether OCT3 inhibitors modulate the effects of MAOi on cardiac function. Administration of an OCT3 inhibitor, CORTI, abolished the effects of MAOi on EF and HR after stimulation with EPI (Fig. 1D, F and Online Table 2). Administration of another OCT3 inhibitor, decynium-22 (200 μg/kg, *i.p* [17]), also suppressed the cardiac responses to EPI and MAOi treatments (Online Figure IIA, B and Online Table 4).

**Fig. 2:**
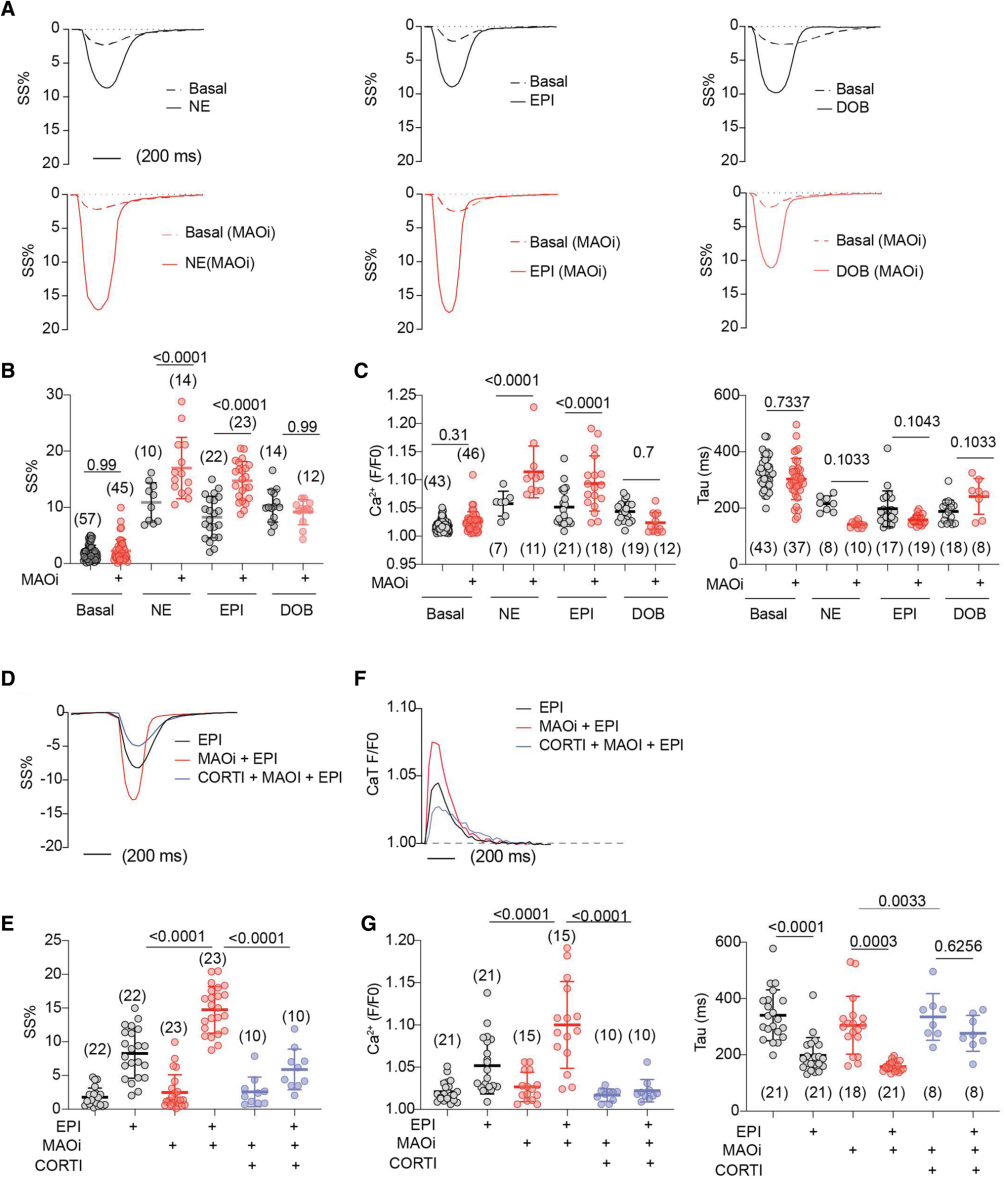
Corticosterone prevents MAOi from enhancing catecholamine stimulation of excitation–contraction coupling in WT AVMs. AVMs were loaded with Ca^2+^ indicator (5 µmol/L Fluo-4 AM) and paced at 1 Hz. AVMs was pretreated with MAOi or CORTI for 5 min and followed by 6-min incubation of β-agonists (NE, EPI, or DOB). SS and Ca^2+^ were recorded for 2 min at baseline and for 6 min after agonist stimulation. The maximal SS and Ca^2+^ were reported. **A**, **B** Representative and quantification of SS% kinetics at the baselines and after stimulation with NE, EPI, or DOB in the absence or presence of MAOi. Dot plots represent the mean ± SD of the indicated number of AVMs from 6 WT mice. **C** Ca^2+^ transient (CaT) amplitude and decay (Tau) in response to NE, EPI, or DOB in the absence or presence MAOi. **D**, **E** Representative and quantification of SS% in response to EPI, MAOi, and CORTI cotreatment. Dot plots represent the mean ± SD of the indicated number of AVMs from 6 WT mice. **F**, **G** Representative CaT dynamics and quantification of CaT amplitude and Tau after EPI, MAOi, and CORTI cotreatment. Dot plots represent the mean ± SD of the indicated number of AVMs from 6 mice. NE = 0.1 µmol/L, EPI = 1 µmol/L, DOB = 1 µmol/L, MAOi = 5 µmol/L, and CORTI = 2 µmol/L. Data are shown as mean ± SD. *p* values were obtained by one-way ANOVA followed with Tukey’s test

To exclude the impacts of CORTI and MAOi on the central nervous system in the observations above, we tested the direct impacts of CORTI and MAOi on isolated AVMs. MAOi enhanced the catecholamine (EPI and NE)-induced sarcomere shortening (SS%) in AVMs, but did not affect the responses to DOB stimulation (Fig. 2A, B). MAOi also enhanced the EPI- and NE-induced Ca^2+^ transient (CaT) amplitude and decay without affecting the responses to DOB (Fig. 2C, D and Online Figure III). Moreover, the effects of MAOi on myocyte contractile and Ca^2+^ responses to EPI stimulation were abolished by CORTI (Fig. 2D–G). Notably, CORTI and anti-depressant MAOi are widely used in clinical settings [51]. These data highlight the interaction between anti-inflammatory CORTI and anti-depressant MAOi in modulating contractile function in the myocardium.

### OCT3 and MAO-A control the accessibility of catecholamines to activate SR-β_1_AR signaling in AVMs

**Fig. 3:**
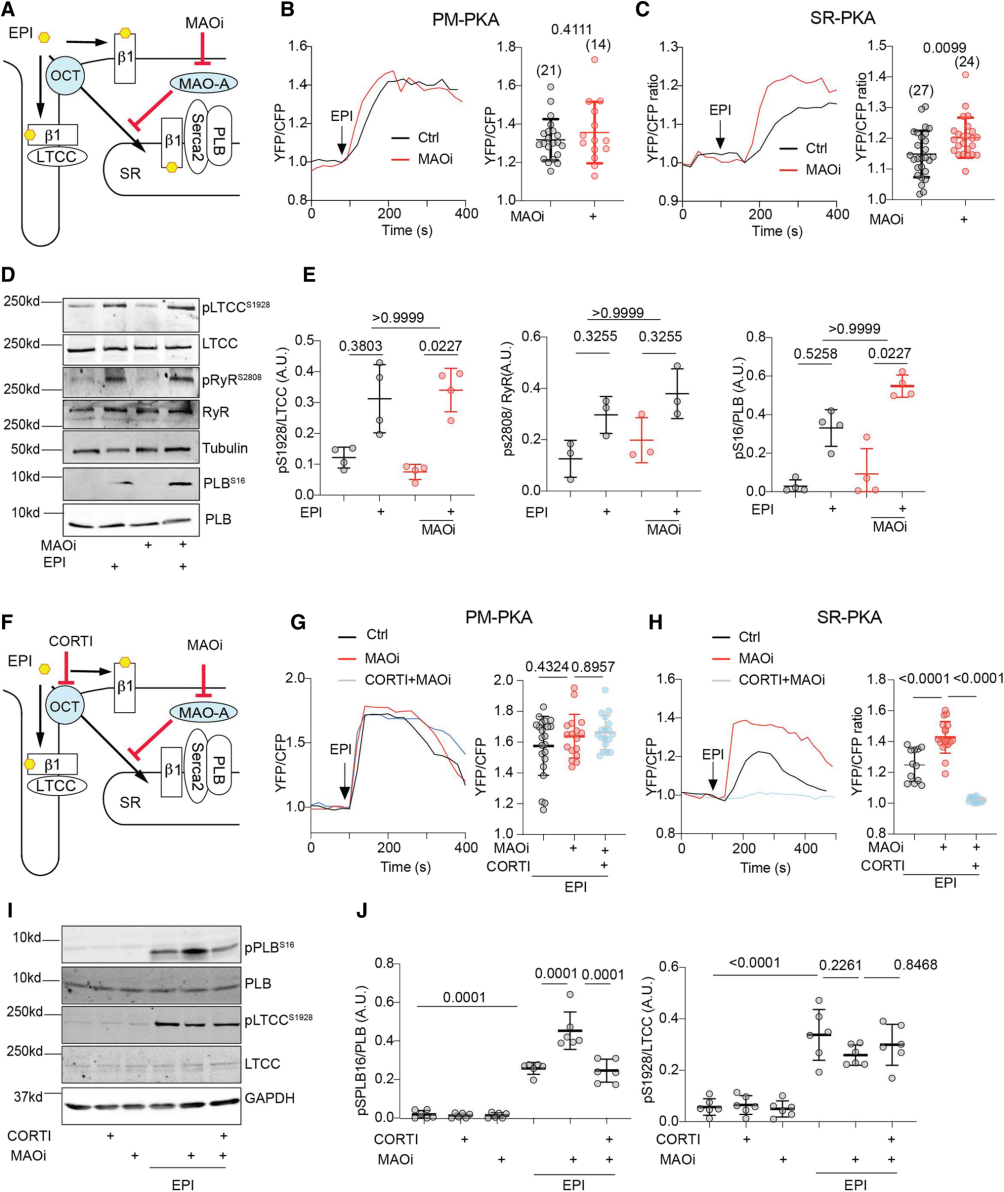
Corticosterone antagonizes the positive effects of MAOi on promoting SR-localized β_1_AR signaling and PLB phosphorylation. Mouse AVMs expressing AKAR3 biosensors were treated with drugs as indicated. YFP/CFP FRET ratio was recorded before and after agonist stimulation for total 400 s. The maximal FRET response after drug stimulation was plotted. AVMs was pretreated with MAOi or CORTI for 5 min and followed by 5-min incubation of β-agonists (EPI or DOB) for western blot. **A** Schematic of intracellular catecholamine hemostasis and local β_1_AR activation is modulated by OCT3 and MAO-A. **B**, **C** WT AVMs were stimulated with EPI after pretreatment with vehicle control (Ctrl) or MAOi. Time courses of FRET dynamics and quantification of local PKA activities at the PM and SR after EPI and MAOi administration were plotted. Dot plots represent the mean ± SD of the indicated number of AVMs from 4 WT mice. **D**, **E** Immunoblots show detection of phosphorylated Ca^2+^ handling proteins (p-Ser 16 of PLB, p-Ser 1928 of LTCC, and p-Ser 2808 of RyR2) in response to EPI in the presence of MAOi. *n* ≥ 3 WT mice. Data were shown as mean ± SD. **F** Schematic depicting the effects of CORTI on subcellular β_1_AR activation by inhibiting OCT3. **G**, **H** Time-course curves and quantification of local PKA activity at the PM and SR after EPI, MAOi, and CORTI coadministration were plotted. Dot plots represent the mean ± SD of the indicated number of AVMs from 4 WT mice. **I**, **J** Immunoblots show detection of phosphorylated LTCC (p-Ser 1928) and PLB (p-Ser 16) in response to coadministration of EPI, MAOi, and CORTI. *n* = 6 rabbits. Data were shown as mean ± SD. EPI = 1 µmol/L, MAOi = 5 µmol/L, and CORTI = 2 µmol/L. A.U. = arbitrary unit. *p* values were obtained by unpaired Student’s *t* test (**B**, **C**), non-parametric Kruskal–Wallis test followed with Dunn’s multiple comparisons test (**E**), or one-way ANOVA followed with Tukey’s test (**G**, **H**, **J**)

Mechanistically, we hypothesized that OCT3 and MAO-A coordinate the activation of the SR-β_1_AR and PKA phosphorylation of PLB at Ser16 by controlling the levels of intracellular catecholamines. In this paradigm, catecholamines enter the cell through OCT3 and are subjected to MAO-A-mediated oxidation and inactivation (Fig. 3A and [57]). To validate the role of the β_1_AR in this regulation, we tested cardiac response in the β_1_AR-KO mice [62] (Online Figure IIC, D, Online Table 5). Deleting β_1_AR abolished the inotropic effects of EPI and MAOi, suggesting that the effects are mainly mediated by the β_1_AR. We then applied a pair of fluorescence resonance energy transfer (FRET)-based biosensors (A-kinase activity reporter 3, AKAR3) anchoring at either the PM or SR microdomains to monitor the local β_1_AR-PKA signaling in AVMs [2, 44]. Inhibiting MAO-A by clorgyline augmented the EPI-induced PKA activity at the SR without affecting the PKA activity at the PM (Fig. 3B, C). The β_1_AR-specific antagonist CGP20712a (0.3 µmol/L) but not the β_2_AR-specific antagonist ICI118551 (1 µmol/L) abolished the SR-PKA FRET response induced by EPI in the presence of MAOi (Online Figure IV), confirming that the β_1_AR is responsible for the effects of MAOi. In agreement, MAOi enhanced the EPI-induced PKA phosphorylation of PLB at Ser16 at the SR, but did not affect the EPI-induced PKA phosphorylation of LTCC at Ser1928 and RyR2 at Ser2808 on the PM (Fig. 3D, E). In controls, MAOi did not affect the PKA phosphorylation of PLB, LTCC, and RyR2 in response to DOB stimulation (Online Figure VA–C).

OCT3 transports catecholamines into myocytes. Thus, inhibition of OCT3 should prevent catecholamine from activating the SR-β_1_AR and suppress the responses to EPI stimulation in AVMs (Fig. 3F and [57, 61]). While MAOi enhanced the SR-PKA activity triggered by EPI in AVMs, CORTI suppressed the SR-PKA response (Fig. 3H). Accordingly, MAOi enhanced the EPI-induced PKA phosphorylation of PLB in AVMs, and the effects of MAOi were attenuated by CORTI (Fig. 3I, J). In comparison, neither MAOi nor CORTI affected the EPI-induced PKA activity and the PKA phosphorylation of LTCC at the PM in AVMs (Fig. 3B, G, I, and J). As controls, neither MAOi nor CORTI affected the PKA activity and phosphorylation of PLB and LTCC after stimulation with DOB (Online Figure VD, E). Our data highlight the essential role of OCT3-dependent entry of catecholamines for activation of the SR-β_1_AR, which is subjected to additional regulation by MAO-A.

### Deletion of MAO-A enhances the local β_1_AR signaling at the SR and promotes E–C coupling and cardiac contraction

**Fig. 4:**
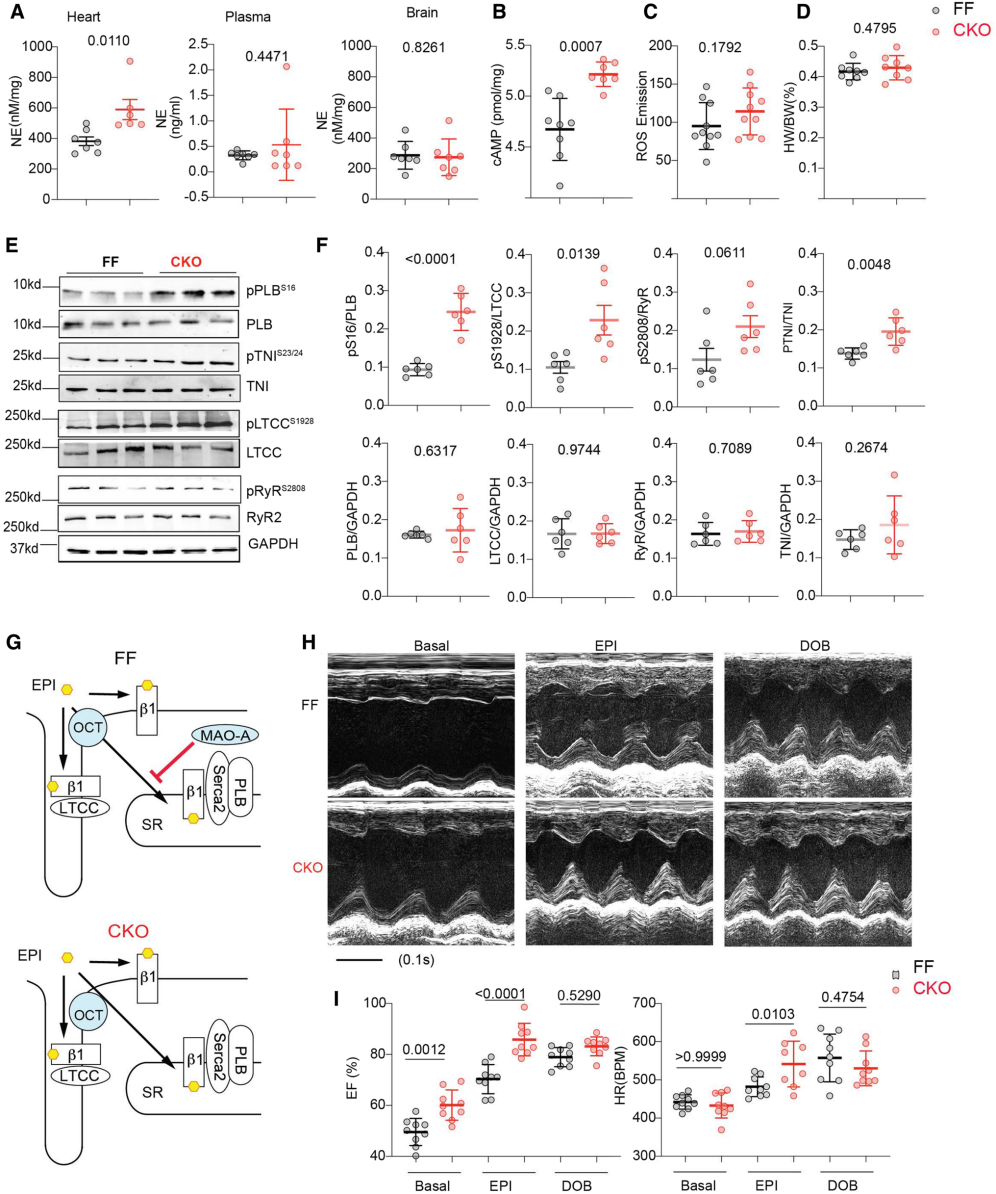
Cardiac-specific deletion of MAO-A increases myocardium catecholamine contents and enhances contractile function. **A** Quantitative measurements of endogenous NE in mouse heart, plasma, and brain (*n* ≥ 6 mice per group). **B** Quantification of cyclic AMP levels in MAO-A-FF and CKO heart tissues (*n* = 8 FF, 7 CKO). **C** Quantification of ROS in mice hearts (*n* = 10 mice per group). **D** Heart weight/body weight ratio in MAO-A-FF and CKO hearts. n = 8. **E**, **F** Western blots show the phosphorylation of PLB (p-Ser16), TnI (p-Ser23/24), RyR2 (p-Ser2808), and LTCC (p-Ser1928) in MAO-A-FF and CKO heart tissues (*n* = 6/group). **G** Schematic shows that deletion of MAO-A enhances local β_1_AR signaling at the SR but not PM microdomain. **H** M-mode echocardiography was recorded for 2 min at baselines and for 8 min after each drug administration. The maximal cardiac response was reported. Representative echocardiography images of MAO-A-FF and CKO mice before and after EPI and DOB stimulation (100 µg/kg, *i.p.*). **I** Quantification of EF and HR before and after EPI or DOB stimulation *n* = 9. Data are shown as mean ± SD. *p* values were obtained by unpaired Student’s *t* test (**A–D**, and **F**) or one-way ANOVA with Tukey’s multiple comparison correction

We further applied cardiac-specific deletion of MAO-A (Online Figure VIA, B) to verify the effects of MAOi in regulating the intracellular SR-β_1_AR signaling and contractile function. MAO-A-CKO mice displayed increased catecholamine concentrations in the myocardium but not in the plasma or brain (Fig. 4A). Consistently, MAO-A-CKO hearts exhibited increases in cAMP and PKA phosphorylation of PLB (Ser16), TnI (Ser23/24), and LTCC (Ser1928) (Fig. 4B, E, and F). Meanwhile, MAO-A displayed a proximity to PLB in AVMs, supporting a local and preferential effect on the SR-β_1_AR signaling (Online Figure VIC, D). Although catecholamine oxidation by MAO-A is known to generate reactive oxygen species (ROS) [26, 27], deleting MAO-A did not change the ROS levels in the myocardium (Fig. 4C), suggesting that ROS unlikely contributes to the enhanced adrenergic signaling in MAO-A-CKO hearts. MAO-A-CKO mice had a similar heart weight/body weight ratio to the FF control littermates (Fig. 4D). MAO-A-CKO mice displayed a modest increase in cardiac EF at the baselines, consistent with the elevated PKA phosphorylation of substrates. When challenged with EPI, MAO-A-CKO mice showed more robust inotropic and chronotropic responses relative to the FF controls (Fig. 4H, I, Online Figure VIE–G, and Online Table 6). In controls, DOB stimulation promoted similar increases in EF and HR in MAO-A-CKO and FF groups (Fig. 4H, I and Online Table 6). Together, these data confirmed that cardiac deletion of MAO-A reduces catecholamine oxidation and inactivation (Fig. 4G) and mimics the effects of MAOi in promoting inotropic and chronotropic responses.

**Fig. 5:**
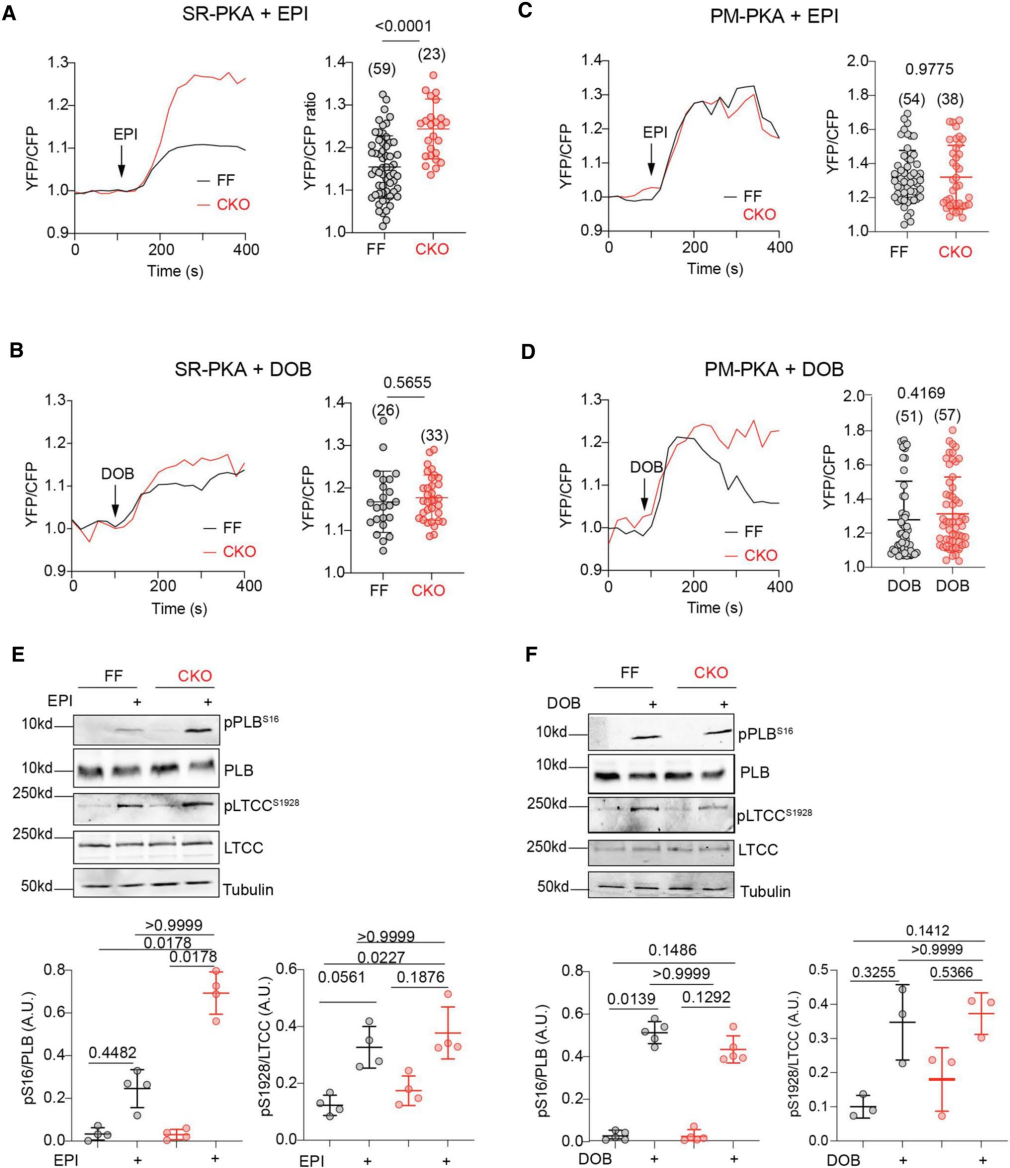
Cardiac-specific deletion of MAO-A selectively enhances PKA signals at the SR microdomain. MAO-A-FF and CKO AVMs expressing AKAR3 biosensors were treated with drugs as indicated. YFP/CFP FRET ratio was recorded before and after agonist stimulation for total 400 s. **A**–**D** Time courses of SR-PKA or PM-PKA FRET ratio changes after 1 µmol/L EPI (top) or DOB (bottom) stimulation in MAO-A-FF and CKO AVMs. The maximal increases in SR-PKA and PM-PKA in AVMs were plotted as mean ± SD of the indicated number of AVMs from 4 MAO-A-FF and 5 CKO mice. **E**, **F** Western blots show detection and quantification of p-Ser 16 of PLB and p-Ser 1928 of LTCC in isolated AVMs after EPI and DOB stimulation (1 µmol/L, 5 min). Dot plots represent mean ± SD of at least three independent experiments in AVMs from MAO-A-FF and CKO mice. Data were shown as mean ± SD. *p* values were obtained by unpaired Student’s *t* test (**A**–**D**) or non-parametric Kruskal–Wallis test followed by Dunn’s multiple comparisons test (**E**, **F**)

**Fig. 6:**
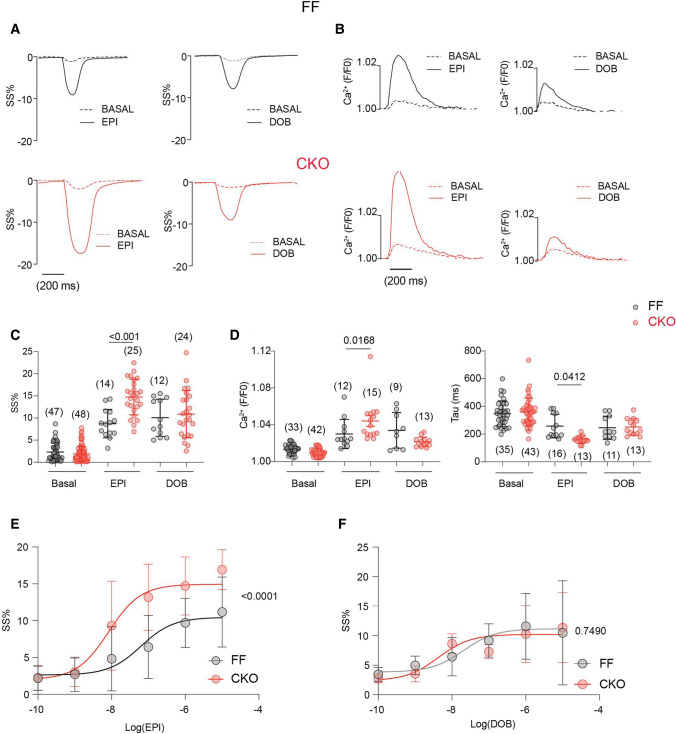
Deletion of MAO-A sensitizes myocyte E–C coupling response to catecholamine. MAO-A-FF and CKO AVMs were loaded with Ca^2+^ indicator, 5 µmol/L Fluo-4 AM, and paced at 1 Hz. AVMs SS and Ca^2+^ were recorded at the baseline and for 6 min after agonist stimulation. The maximal response was reported. **A**, **C** Representative and quantification of SS% at the baseline and after stimulation with 1 µmol/L EPI or DOB. Dot plots represent the mean ± SD of the indicated number of AVMs from 4 MAO-A-FF and 6 CKO mice. **B**, **D** Representative traces and quantification of CaT and Tau at the baseline and after stimulation with 1 µmol/L EPI or DOB. Dot plots represent the mean ± SD of the indicated number of AVMs from 4 MAO-A-FF and 6 CKO mice. **E**, **F** Agonist dose–response curves of SS% to EPI (EC50, CKO 82.9 ± 12.9 nmol/L, FF 62.9 ± 7.8 nmol/L) or DOB (EC50, CKO 42.5 ± 7.9 nmol/L, FF 20.8 ± 7.3 nmol/L) stimulation were analyzed in MAO-A-FF and CKO AVMs. Data represent the mean ± SD of the AVMs from 6 MAO-A-FF and 8 CKO mice. *p* values were obtained by one-way ANOVA (**C**, **D**) or two-way ANOVA (**E**, **F**) test followed by Tukey’s multiple comparison correction

At the cellular levels, EPI triggered rapid increases in the PM-PKA and SR-PKA activity in both MAO-A FF and CKO AVMs (Fig. 5A, C). Compared to the FF group, MAO-A-CKO AVMs had higher PKA activities at the SR and a left-shifted dose–response curve of SR-PKA activity induced by EPI (Fig. 5A, C and Online Figure VIIA). Deleting MAO-A, however, did not affect the dose–response curve of PM-PKA activity induced by EPI (Online Figure VIIB). Consequently, deleting MAO-A enhanced the EPI-induced phosphorylation of PLB at Ser16 without affecting the PKA phosphorylation of LTCC (Fig. 5E, F). EPI also promoted larger increases in SS% in MAO-A-CKO AVMs relative to the FF controls, associated with higher Ca^2+^ transient amplitudes and faster Ca^2+^ decay tau (Fig. 6A–E). In contrast, deleting MAO-A did not affect the DOB-induced PKA responses at either the PM or SR microdomains (Fig. 5B–D and Online Figure VIIC, D). Deleting MAO-A did not affect the DOB-induced responses in PKA phosphorylation, myocyte shortening, and Ca^2+^ transient, either (Figs. 5E, F and 6A–E). Together, these results confirm that MAO-A deletion enhances the SR-β_1_AR signaling and E–C coupling by attenuating catecholamine oxidation.

Conversely, overexpressing MAO-A attenuated the SR-PKA activity and right-shifted the concentration–response curves of the SR-PKA activity after NE or EPI stimulation without affecting the PM-PKA (Online Figure VIIIA, B). MAO-A over-expression, however, did not affect the subcellular PKA activity in response to DOB stimulation (Online Figure VIIIA, B). Accordingly, MAO-A over-expression attenuated the PKA phosphorylation of PLB at ser16 but not LTCC at ser1928 induced by NE or EPI. In contrast, MAO-A over-expression did not affect the PKA-dependent phosphorylation induced by DOB (Online Figure VIIIC–E). Thus, an elevated MAO-A selectively inhibits the β_1_AR-PKA signaling and substrate phosphorylation at the SR in response to catecholamines.

### OCT3 inhibition impairs the MAO-A-dependent augments of intracellular SR-β_1_AR signaling and cardiac contractility

**Fig. 7:**
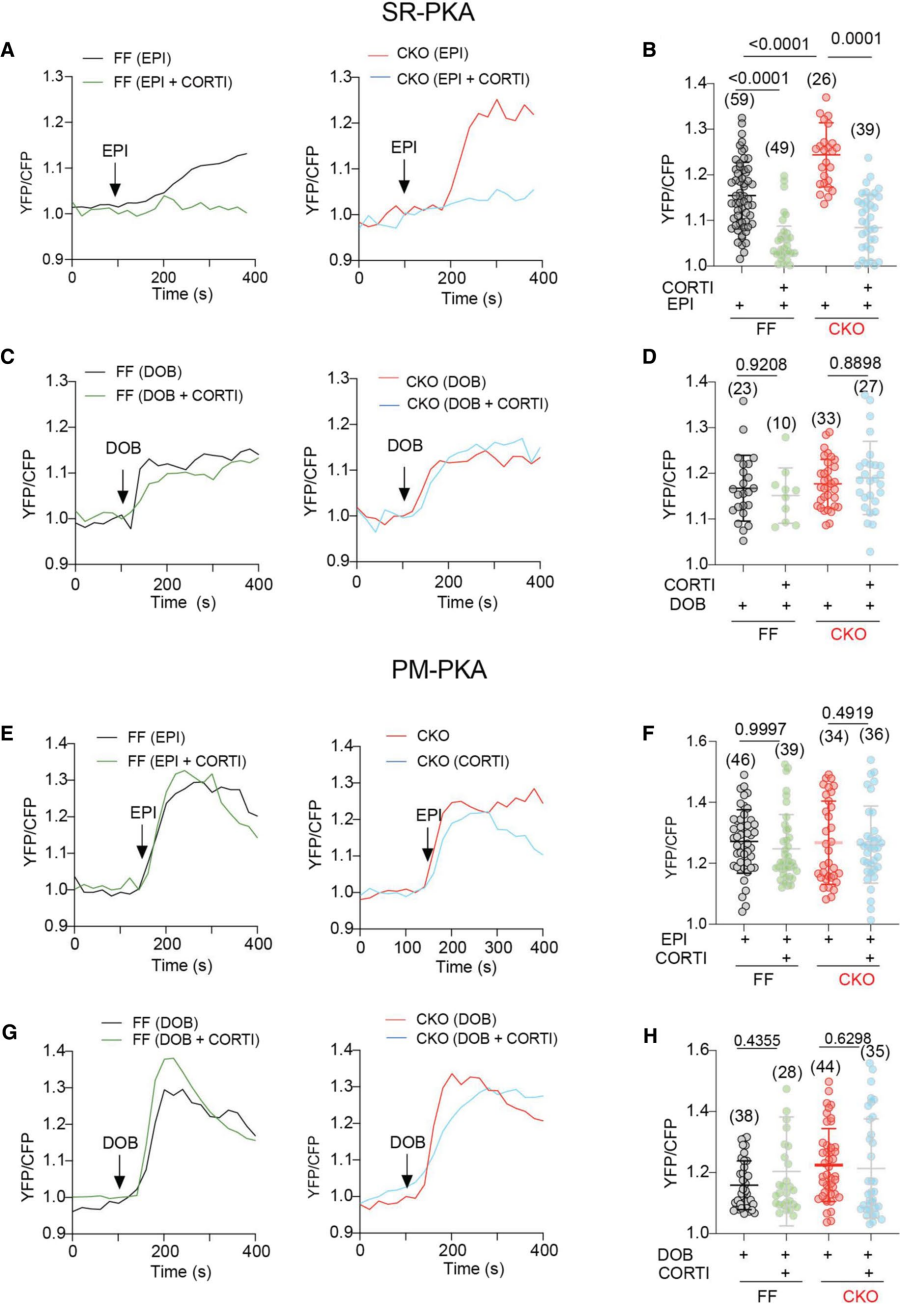
Inhibiting OCT3 by corticosterone abrogates the amplification of SR-localized β_1_AR signaling in MAO-A-CKO. MAO-A-FF and CKO AVMs expressing AKAR3 biosensors were treated with drugs as indicated. YFP/CFP FRET ratio was recorded before and after agonist stimulation for total 400 s. **A**–**D** Time courses and quantification of changes in SR-PKA FRET ratio after EPI or DOB (1 µmol/L) stimulation in the presence of CORTI pretreatment (2 µmol/L, 5 min). **E**–**H** Time courses and quantification of changes in PM-PKA FRET ratio after EPI or DOB (1 µmol/L) stimulation in the presence of CORTI pretreatment (2 µmol/L, 5 min). Dot plots represent the mean ± SD of the indicated number of MAO-A-FF and CKO AVMs from 4 MAO-A-FF and 5 CKO mice. Data were shown as mean ± SD. *p* values were obtained by one-way ANOVA test followed by Tukey’s test

**Fig. 8:**
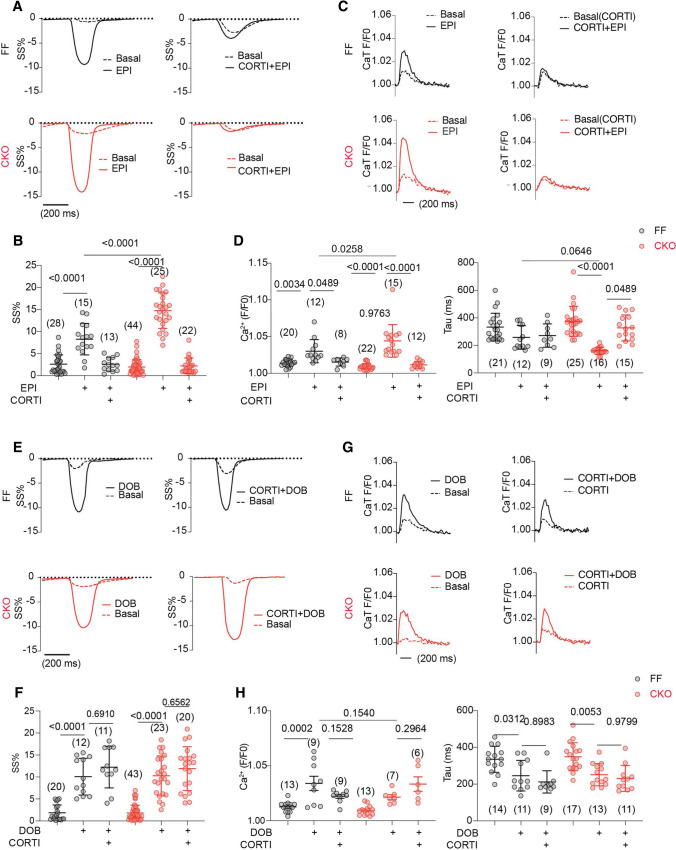
Corticosterone abolishes the efficacy of MAO-A deletion in promoting contractility and Ca^2+^ cycling. AVMs were incubated with 5 µmol/L Fluo-4 AM and paced at 1 Hz. SS and Ca^2+^ were recorded at baseline and for 6 min after agonist stimulation. The maximal SS and Ca^2+^ responses were reported. **A**, **B** Representative traces and maximal increases in SS% after EPI (1 µmol/L) stimulation with or without CORTI pretreatment (5 min). **C**, **D** Ca^2+^ dynamics and quantification of CaT and Tau before and after EPI (1 µmol/L) stimulation with or without CORTI pretreatment (2 µmol/L, 5 min). Dot plots represent the mean ± SD of the indicated number of AVMs 4 MAO-A-FF and 7 CKO mice. **E**, **F** Representative SS% curve and maximal SS% before and after DOB (1 µmol/L) stimulation with or without CORTI pretreatment (5 min). **G**, **H** Ca^2+^ dynamics and quantification of CaT and Tau before/after DOB (1 µmol/L) stimulation with or without CORT pretreatment (2 µmol/L, 5 min). Dot plots represent the mean ± SD of the indicated number of AVMs 4 MAO-A-FF and 7 CKO mice. *p* values were obtained by one-way ANOVA followed by Tukey’s test

While EPI stimulation induced the higher SR-PKA response in MAO-A-CKO AVMs relative to FF controls, inhibiting OCT3 by CORTI reduced the SR-PKA responses in both genotypes (Fig. 7A, B). In comparison, the PKA activity at the PM was comparable between MAO-A-CKO and FF AVMs, and was not affected by CORTI (Fig. 7E, F). While MAO-A-CKO AVMs exhibited higher increases in SS% and Ca^2+^ transient amplitude and faster Ca^2+^ decay tau after EPI stimulation, CORTI abrogated the these increases in both genotypes (Fig. 8A–D). In contrast, CORTI did not affect the PKA activity at either the PM or SR in responding to DOB (Fig. 7C, D, G, H) as DOB crosses the cell membrane independent of OCT3 (https://pubchem.ncbi.nlm.nih.gov). Moreover, the DOB-induced contractile and Ca^2+^ responses were not affected by CORTI in either MAO-A-CKO or FF AVMs (Fig. 8E–H).

**Fig. 9:**
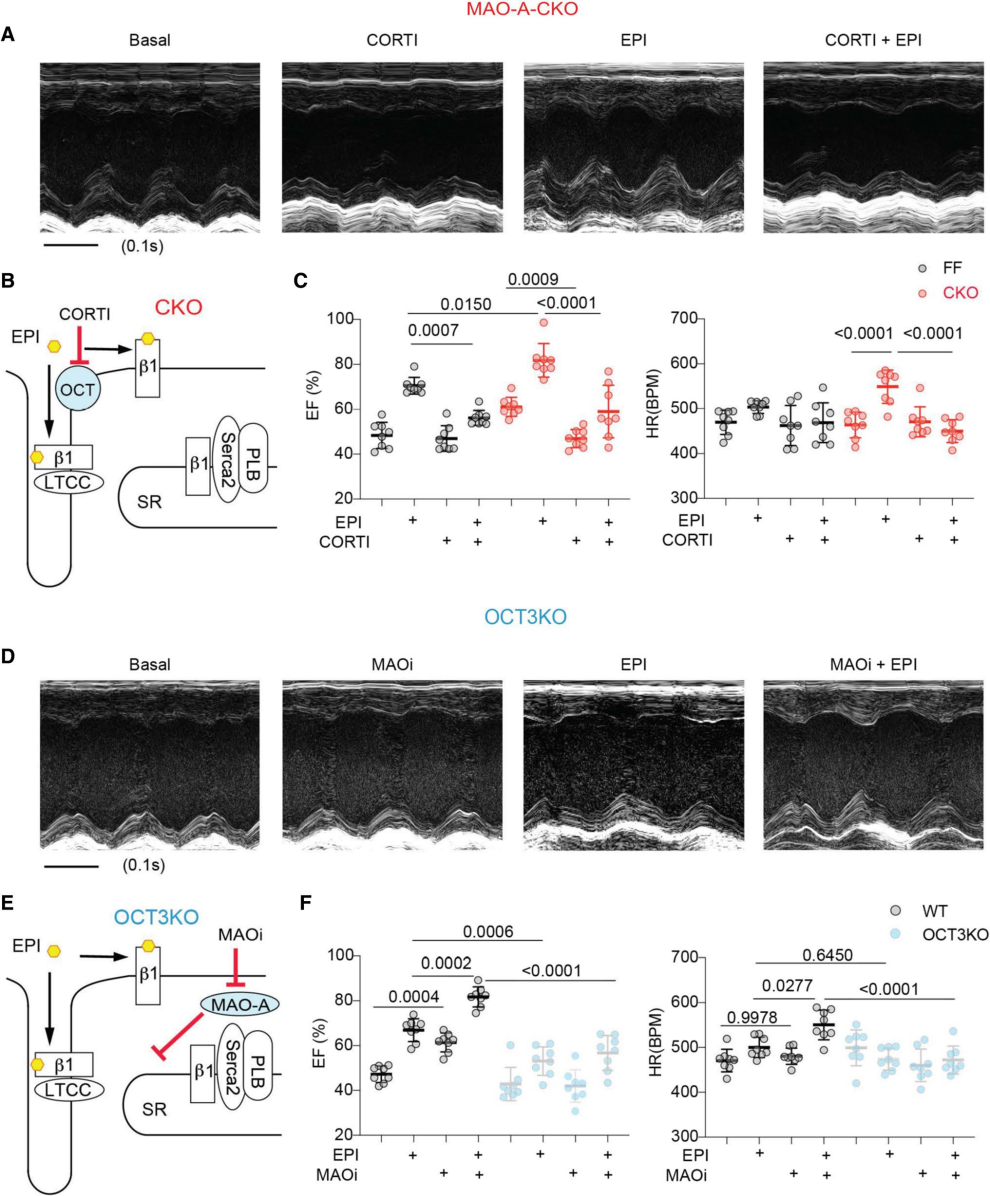
MAO-A and OCT3 coordinate cardiac fight-or-flight response. M-mode echocardiography was recorded for 2 min at baselines and for 8 min after drug administration. The maximal cardiac response was reported. **A** Representative echocardiography images of MAO-A-CKO mouse heart before and after EPI stimulation (100 µg/kg, *i.p*.) with and without CORTI pretreatment (200 µg/kg, *i.p*., 5 min). **B** The schematic depicts that inhibiting OCT3 by CORTI suppresses the SR-localized β_1_ARs activation in MAO-A-CKO hearts. **C** The EF and HR of MAOA-A-FF and CKO mice before and after treated with EPI and CORTI described in **A**. n = 8 mice in each condition. **D** Representative echocardiography images of OCT3-KO mouse heart before and after EPI stimulation (100 µg/kg, *i.p*.) with and without MAOi (0.4 mg/kg, *i.p*.). **E** The schematic depicts that deletion of OCT3 prevents the effects of MAOi on enhancing the SR-localized β_1_AR signaling. **F** The EF and HR in WT and OCT3-KO mice before and after EPI and MAOi treatment described in **D**. *n* = 8 KO mice. Data were shown as mean ± SD. *p* values were obtained by one-way ANOVA followed by Tukey’s test

We then validated the functional interaction between OCT3 and MAO-A using MAO-A CKO and OCT3-KO mice in vivo. While EPI triggered stronger increases in EF and HR in MAO-A CKO mice relative to FF controls, CORTI attenuated the inotropic and chronotropic responses in both genotypes (Fig. 9A–C and Online Table 7). Conversely, administration of EPI modestly enhanced cardiac EF and HR in OCT3-KO mice; and MAOi failed to improve the cardiac EF response to EPI stimulation (Fig. 9E–H and Online Table 8). Our results reveal that deletion and inhibition of MAO-A enhance the intracellular β_1_AR signaling and cardiac E–C coupling and contractility. Deletion and inhibition of OCT3 abrogate the MAO-A-dependent effects on the intracellular β_1_AR signaling and cardiac function.

Furthermore, we characterized the interaction between MAO-A and OCT3 in mice with ischemic HF induced by myocardial infarction (MI, Online Figure IX). The ischemic HF was indicated by the Sirius Red staining and decreased EF (Online Figure IXA–C and Online Table 9). The MI hearts displayed increased MAO-A expression and reduced β_1_AR expression but no change in OCT3 expression relative to healthy controls, consistent with previous studies (Online Figure IXB, C and [31]). Application of MAOi promoted cardiac EF in the MI mice, whereas inhibiting OCT3 by CORTI prevented the effects of MAOi (Online Figure IXB, C and Online Table 10). These data indicate that the interaction between MAOi and OCT3 is maintained in the ischemic HF.

## Discussion

The sympathetic regulation in the heart is achieved through the catecholamine-activated βARs and downstream cAMP-PKA signaling, which is diminished in cardiac diseases [29, 52]. We have recently identified a functional pool of β_1_ARs associated with SERCA2a on the SR that is essential for promoting SERCA2a function in E–C coupling [57]. In this study, cardiac knockout of MAO-A elevates catecholamine contents, enhances the local β_1_AR-cAMP-PKA signaling and PKA phosphorylation of PLB at the SR, and sensitizes myocardial contractile responsiveness to endogenous and exogenous catecholamines in vivo. However, suppressing catecholamine transport by genetic deletion of OCT3 negates the effects of MAO-A deficiency in promoting the SR-β_1_AR responsiveness and cardiac contraction. These data demonstrate that MAO-A and OCT3 gate the SR local β_1_AR signaling and fine-tune cardiac fight-or-flight responses. Additionally, CORTI, the OCT3 inhibitor, counteracts MAOi in modulating the catecholamine-driven intracellular SR-β_1_AR signaling and cardiac function. Our data thus unravel the contra-indicatory effects of anti-inflammatory CORTI and anti-depressant MAOi in regulating cardiomyocyte catecholamine homeostasis and cardiac contractile response, providing novel implications of those drugs in patients with cardiac diseases. Despite the promising therapeutic application indicated from both in vitro and mice model, systemic clinical trial-like studies using larger animal models, including pig or monkey model, are essential to confirm the safety and efficacy of inhibiting MAO-A or OCT3. For these confirmatory studies, a bigger sample size and pre-registered experimental protocol with a clearly defined endpoint should be deployed to improve the translation of this innovative hypothesis targeting intracellular β_1_-AR for managing heart function. Given this study belongs to the Stage II (hypothesis-generating exploratory studies) of preclinical research [9], the significant *p* value (*p* < 0.05) should be considered as establishing a new hypothesis.

### Heterogeneity of cardiac adrenergic receptor signaling at the cell surface and intracellular membrane

The highly organized architecture in cardiomyocytes provides spatial context for compartmentalized microdomain and local cAMP/PKA signaling in regulating E–C coupling [50, 65]. Increasing evidence supports the key roles of subcellular distribution of βARs in precisely regulating local cAMP/PKA activity in cardiomyocytes [36, 57]. In a classic view, the βARs signal from the PM, including T-tubular and crest membrane. While the β_1_ARs are found on the entire cell surface, the β_2_ARs are exclusively targeted to the T-tubules [3]. The T-tubular membrane forms dyads together with the juxtamembrane of the SR, allowing tight coupling between LTCC and RyR2 and increasing [Ca^2+^]i during E–C coupling. The β_1_ARs and β_2_ARs at these membrane microdomains are critical for the local PKA phosphorylation of LTCC and RyR2 [4, 23, 56]. In comparison, the β_1_ARs at the crest membrane are believed to induce signals to other organelles, such as myofilaments and mitochondria [2]. Recent studies show functional βARs at the intracellular compartments, including the nuclei, the Golgi, and the SR [5, 36, 57]. In particular, the SR-localized b_1_ARs associate with SERCA2a and are critical for promoting the local PKA activity and phosphorylation of PLB and enhancing SERCA2a dependent Ca^2+^ reuptake into the SR [57]. These observations highlight the highly localized subcellular βAR signaling to coordinate E–C coupling machinery. The identification of specific β_1_AR signaling nano-domains at the dyad-associated junctional SR membrane [4] and the SERCA2a-associated longitudinal SR membrane [57] offers opportunities to explore the compartmentalized β_1_AR signaling in physio(patho)logical conditions.

In this study, we show that MAO-A and OCT3 are indispensable players in fine-tuning the catecholamine-induced SR-β_1_AR signaling in the heart. Catecholamines, including NE and EPI, are membrane impermeant and enter the cells via organic cation transporters OCT3 and plasma membrane monoamine transporter (PMAT) [14, 19]. Our data show that OCT3 deletion and inhibition can reduce the SR-b_1_AR signaling in the hearts, whereas the potential role of PMAT in modulating the SR-β_1_AR signaling in hearts remains to be examined. In comparison, many synthetic ligands, including isoproterenol, DOB, and β-blockers (e.g., carvedilol and alprenolol), can cross the membrane and reach the internal β_1_ARs by passive diffusion. Inside cardiomyocytes, catecholamines are subjected to the MAO-mediated oxidative degradation [7]. The SR is physically connected to mitochondria [45]. MAO-A is localized at the mitochondrial outer membrane, which displays proximity to PLB and regulates the SR-β_1_AR signaling in a local vicinity. Inhibition of MAO-A selectively enhances the SR-β_1_AR signaling without affecting the β_1_AR at the PM. Thus, OCT3 and MAO-A act as dual-level gauges to fine-tune intracellular catecholamine levels, the local PKA activity at the SR, and SERCA2a function during physiological stress. This setting could have two benefits: calibrating cardiac contractility according to the intensity of the sympathetic tone (i.e., the catecholamine levels) and avoiding catecholamine storms from overdriving cardiac βARs and subsequent arrhythmia.

### MAO-A differentially modulates PM and intracellular adrenergic receptor signaling in HF

Desensitized βAR signaling and depressed contractile response are hallmarks of HF [6]. The desensitization of βAR signaling is correlated with the reduced β_1_AR density at the PM, catecholamine content, cAMP signal, and PKA activity in failing hearts [6, 27]. However, evidence indicates heterogeneity of PKA phosphorylation of substrates in failing hearts. For example, while the PKA phosphorylation of PLB is commonly reduced in HF patients, the phosphorylation of LTCC and RyR2 may not be changed or even increased [2, 4, 44]. The reason for the differential regulation of these substrates is not entirely understood. Previous studies have shown that the cardiac β_1_AR undergoes translocation from the PM to the intracellular compartments in HF, including increased association with SERCA2a on the SR [58]. However, the increased association between the β_1_AR and SERCA2a in HF fails to enhance the PKA phosphorylation of PLB. Recent studies reveal that despite excessive circulating plasma catecholamines in HF patients, cardiac NE content is decreased and accompanied by diminished NE uptake and elevated MAO-A expression [27, 47]. The increased MAO-A expression prevents the access of catecholamines to the SR-β_1_AR and effectively inhibits the intracellular receptor activity. Additionally, alternations of other signaling molecules, such as phosphodiesterase and phosphatases, also contribute to the remodeling of the local βAR signaling [65]. Moreover, evidence indicates that the up-regulated expressions of other adrenergic receptors, including αAR, β_2_AR, and β_3_AR, regulate cardiac contraction and heart rate in the failing human heart [24, 37, 60, 64]. While our observations are limited to mice and rabbits, future studies should be pursued to dissect the intracellular adrenergic signaling in human heart failure.

### MAO-A underlies cross talk between depression and HF

Multiple dynamic processes regulate catecholamine homeostasis, including synthesis, storage, release, reuptake, and metabolism. Notably, the reuptake and metabolism of catecholamines are key regulators in multiple sympathoadrenal disorders [15, 47]. Impaired homeostasis of catecholamines is a hallmark of sympathetic dysregulation and contributes to the progression of neurological and cardiac diseases [18, 40, 66]. For example, increased MAO-A expression and catecholamine abnormalities are associated with heart diseases [18, 66], Parkinson’s diseases [40], and age-associated impaired lipolysis [41]. Thus, catecholamines occupy key positions in managing health and diseases [15]. Drugs targeting noradrenergic transmission (e.g., MAO inhibitors, inotropes, and β-blockers) are first-line treatments for sympathoadrenal disorders and contemplate the development of new therapeutic strategies [16, 34, 51].

Interestingly, depression is common in HF patients (42% of the patient [32]) and is underrecognized and linked to adverse outcomes, such as poor cardiac function and elevated mortality [8]. Effective treatments for HF patients with depression are urgently needed. However, despite the wide use of MAOi in the central nervous system, the role of MAO-A in the heart is not well understood. MAOi has been shown to increase both central and peripheral catecholamine levels [22], whereas an overdose of MAOi can lead to overaction of the sympathetic nervous system, hypertension, and tachycardia [48, 59]. Recent studies show that cardiac-specific MAO-A over-expression leads to chronic ventricle dysfunction [54]. MAO-A inhibition prevents cardiac remodeling and dysfunction in the pressure-overload-induced HF model [27]. In rodent hearts, MAO-A is also up-regulated in aging and HF and contributes to the increased reactive oxidative species [39]. Inhibition of MAO-A is beneficial in HF by preventing the production of reactive oxidative species. Our data demonstrate that MAOi enhances cardiac adrenergic response via regulating the intracellular βAR signaling, indicating that inhibition of MAO-A may offer additional benefit to the myocardium by enhancing the intracellular adrenergic signaling and rescuing the impaired SERCA2a function. Compared with conventional treatment of inotropes, which do not account for the subcellular localization of βARs and are known to cause oxidative injury and arrhythmias, restoring local catecholamines and subcellular adrenergic sensitivity may have considerable potential and advantages in treating heart diseases.

### Contraindication between anti-inflammation and anti-depressant drugs in the heart

Catecholamine import and degradation by OCT3 and MAO-A determine the local concentration, duration, and physical spread of released catecholamines, critically modulating the magnitude, duration, and diversity of their effects in physio(patho)logical conditions [20]. Given their role in modulating neurotransmitters, MAO-A and OCT3 in the brain are also pathogenic factors of depressive disorders [11, 33, 35, 42, 51]. Here, we found elevated cardiac catecholamine levels in cardiac-specific MAO-A knockout mice. By contrast, OCT3 knockout mice demonstrated decreases in tissue levels of catecholamines in the hearts [53]. While MAO-A inhibition effectively enhances the SR-β_1_AR signaling and PKA phosphorylation of PLB, the efficacy of MAO-A inhibition is abrogated by inhibiting OCT3 with CORTI. These observations again support that a joint mechanism involving MAO-A and OCT3 exists in the central and peripheral disorders. Indeed, fludrocortisone, a corticosteroid similar to CORTI, has been used to antagonize the effect of MAOi in the clinical treatment of neuronal disorders [10]; and a high cortisol level is used as a predictor of nonresponse to MAOi treatment in patients [25]. Moreover, dexamethasone, a synthesized corticosteroid inhibiting OCT3, decreases MAO-A catalytic activity on monoamine substrates [49]. Acute treatment of dexamethasone decreases mouse heart diastolic function, which is highly regulated by SERCA function [1]. Our study thus provides a fresh perspective on the application of MAOi in managing comorbidity of HF and depression. However, cautions remain for MAOi treatment in patients with elevated CORTI levels, given that CORTI undermines the efficacies of MAOi.

In summary, we define MAO-A and OCT3 as essential regulators in calibrating access of catecholamines to the intracellular β_1_AR at the SR and fine-tuning cardiac fight-or-flight response. Our data support the potential utility of anti-depressant MAOi in rescuing β_1_AR signaling and inotropic responses in HF, whereas CORTI may exacerbate depressed cardiac ejection fraction.

## Supplementary Information

Below is the link to the electronic supplementary material.

Supplementary file 1 (PDF 1754 KB)

Supplementary file 2 (DOCX 51 KB)

Supplementary file 3 (DOCX 59 KB)

## Data Availability

All experimental reagents used in the study are commercially available (see Online Methods). All raw data generated or analyzed during this study are included in this published article (and its supplementary files). All other data or resources are available from the corresponding author upon request.
