# Supplementary material for: Monoamine oxidase A and organic cation transporter 3 coordinate intracellular β1AR signaling to calibrate cardiac contractile function
Source: Basic Res Cardiol. 2022 Jul 17;117(1):37. doi: 10.1007/s00395-022-00944-5 (PMC9288959; doi:10.1007/s00395-022-00944-5)

Online Figure I

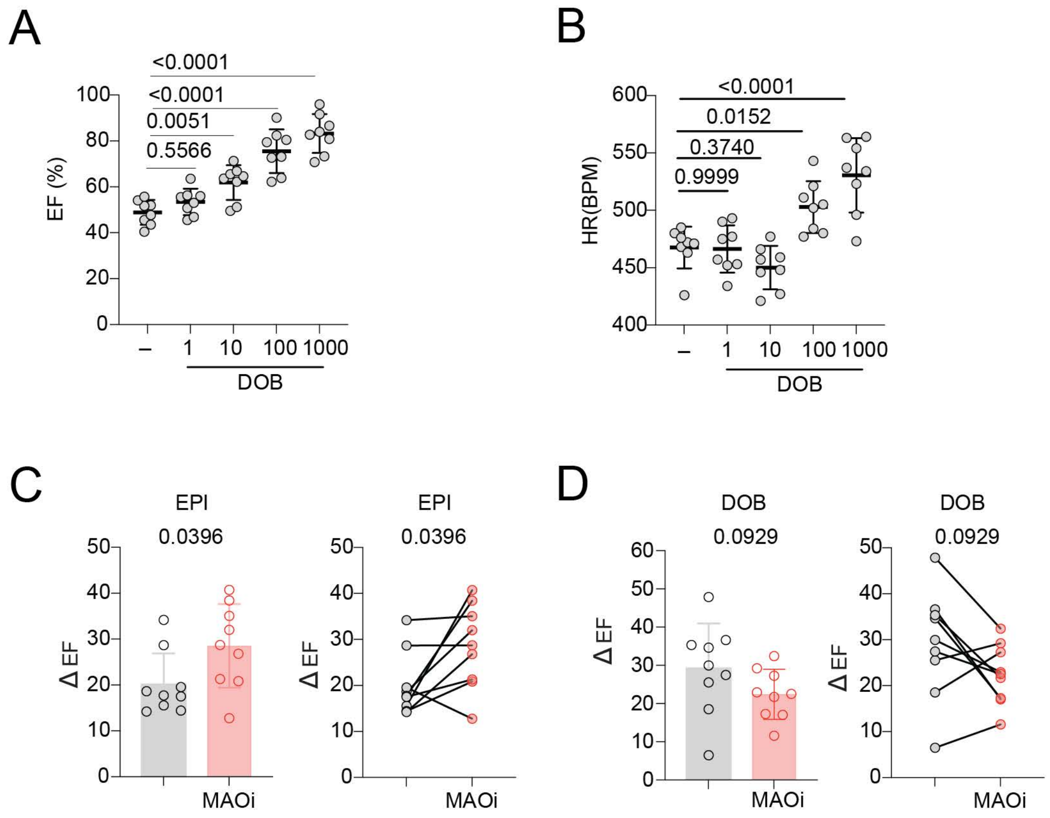

Online Figure II

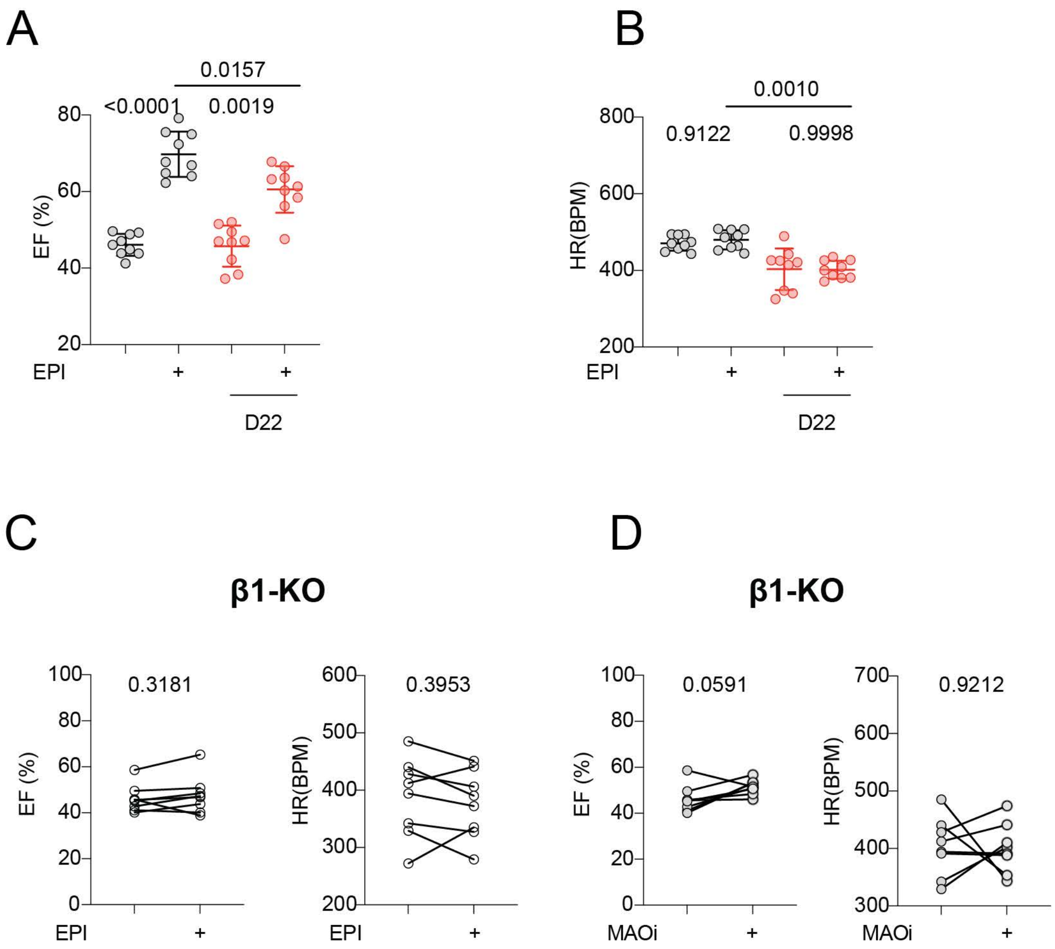

Online Figure III

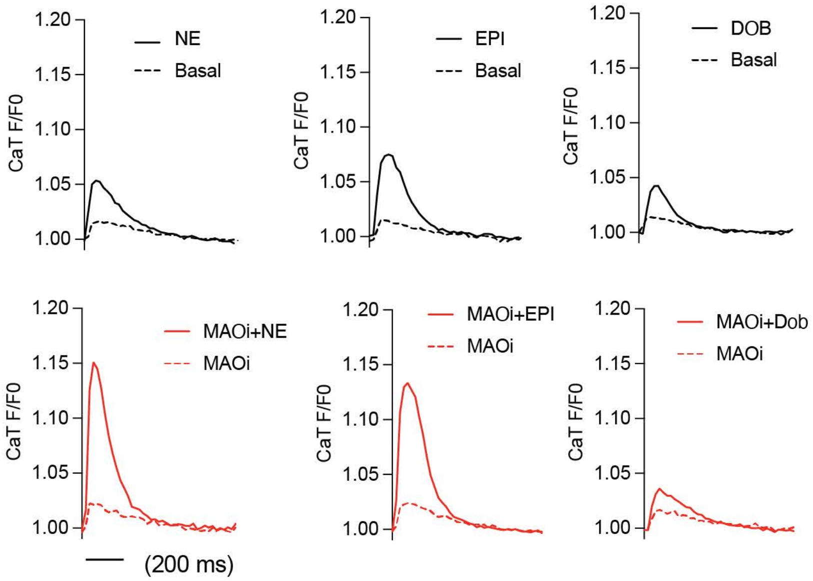

Online Figure IV

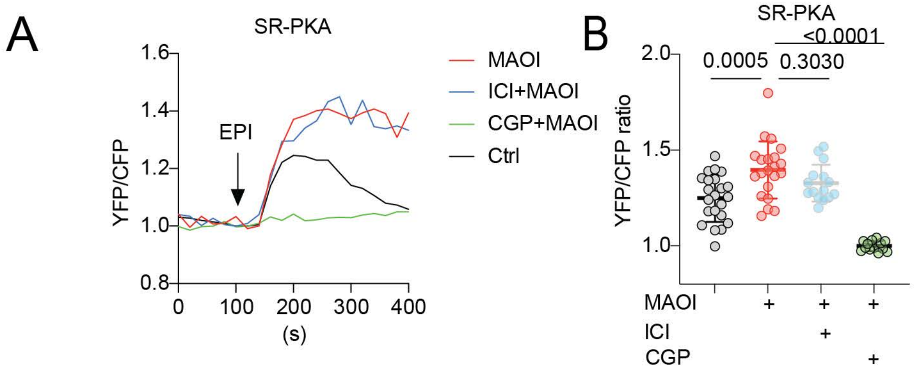

Online Figure V

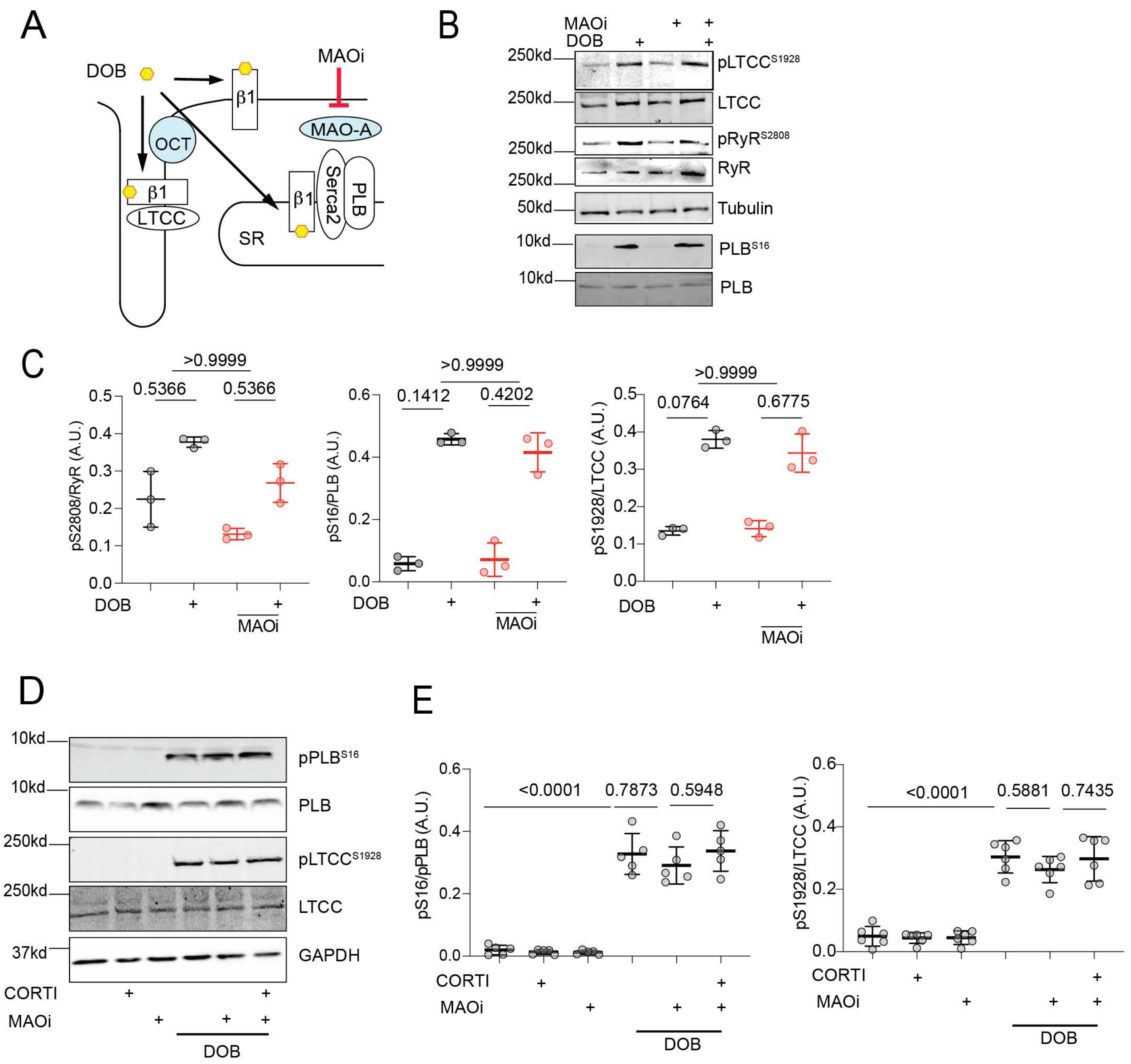

Online Figure VI

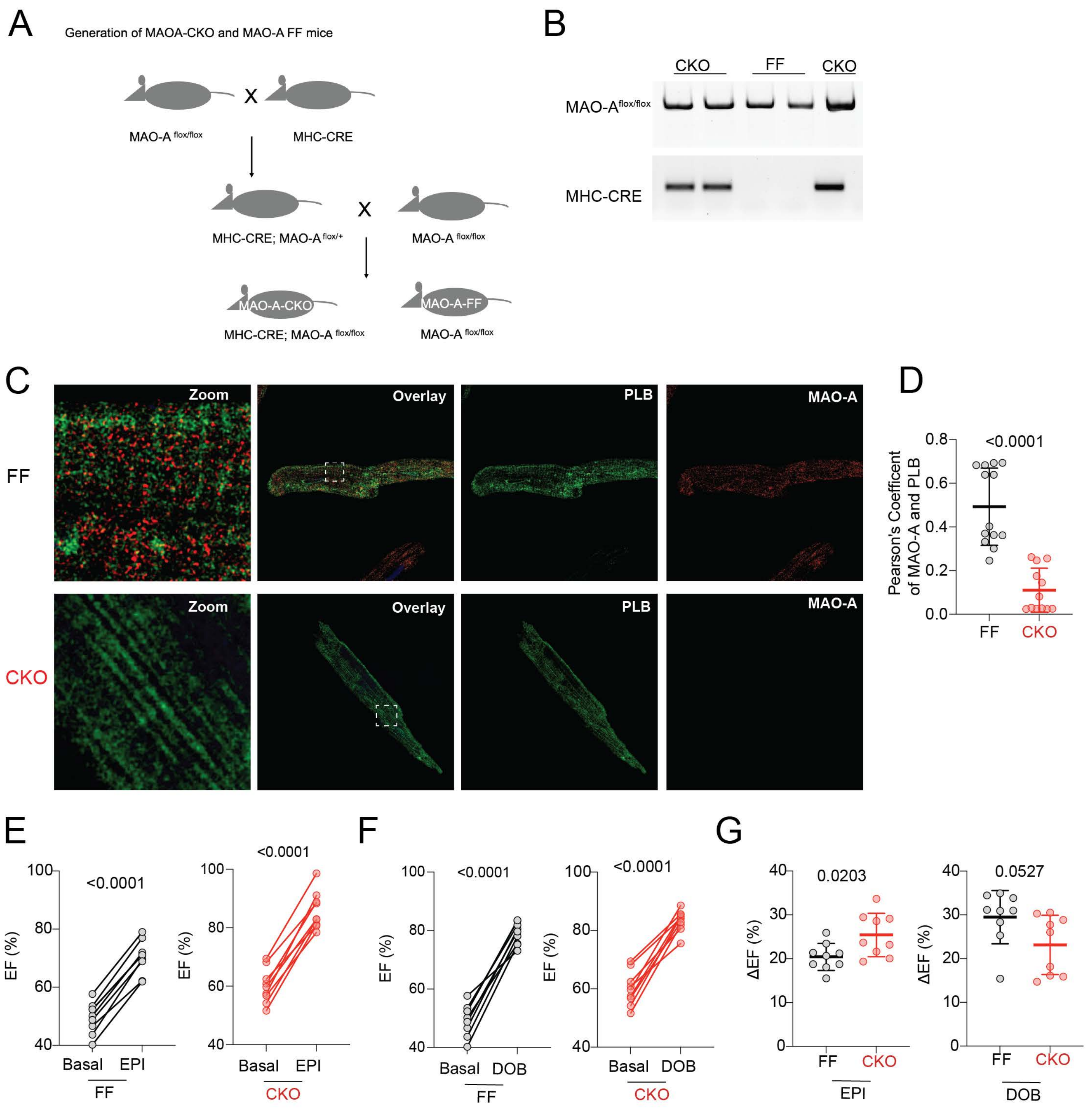

Online Figure VII

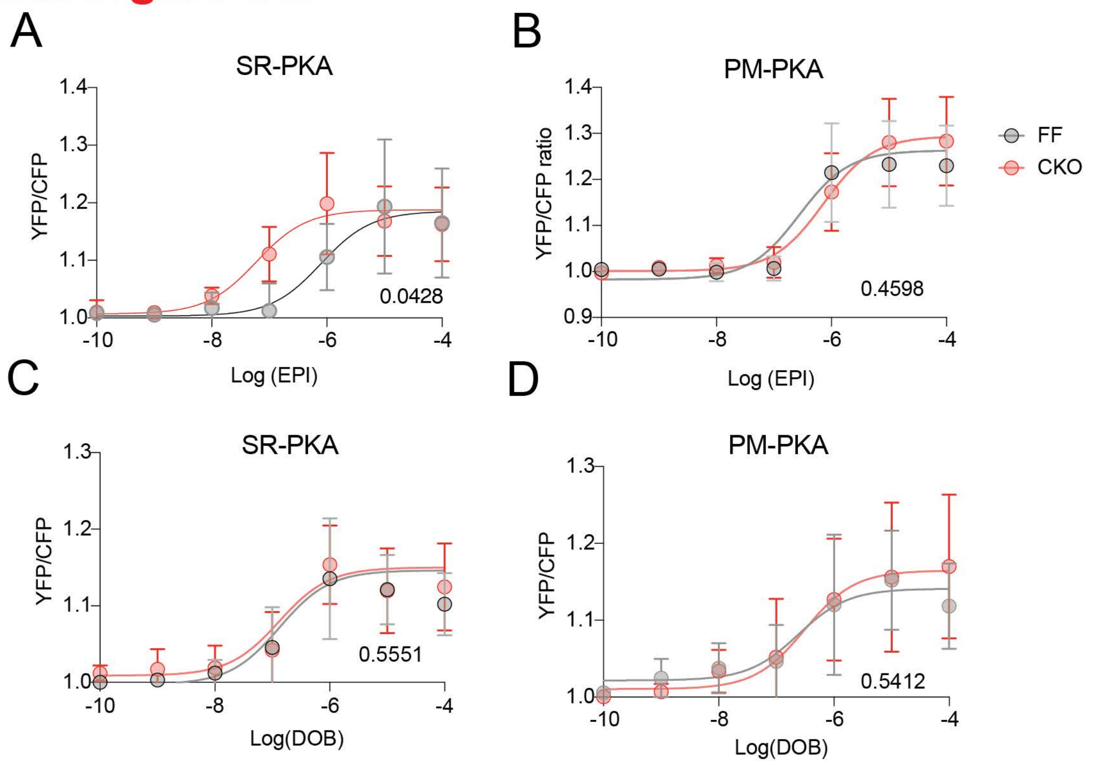

Online Figure VIII

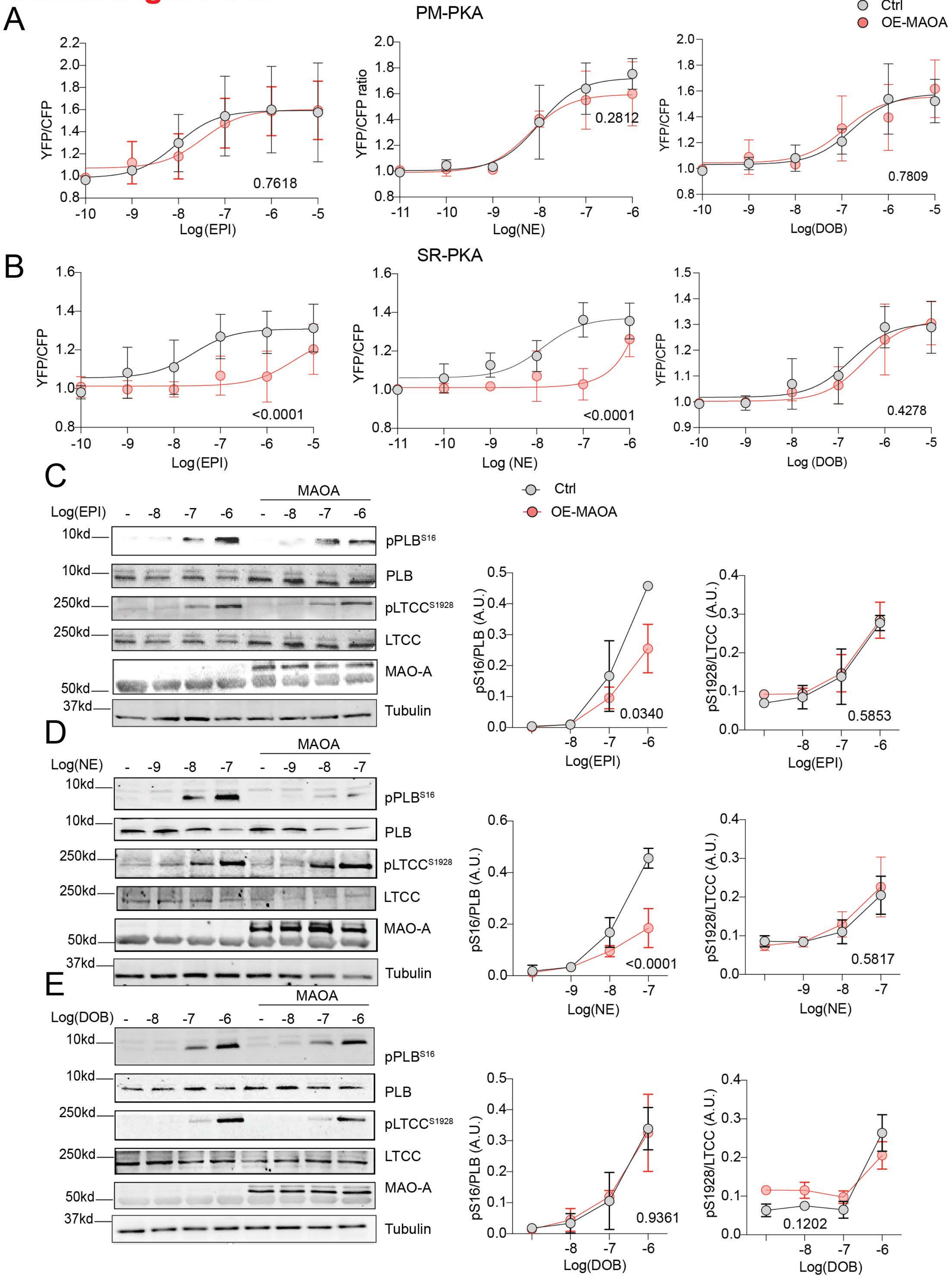

Online Figure IX

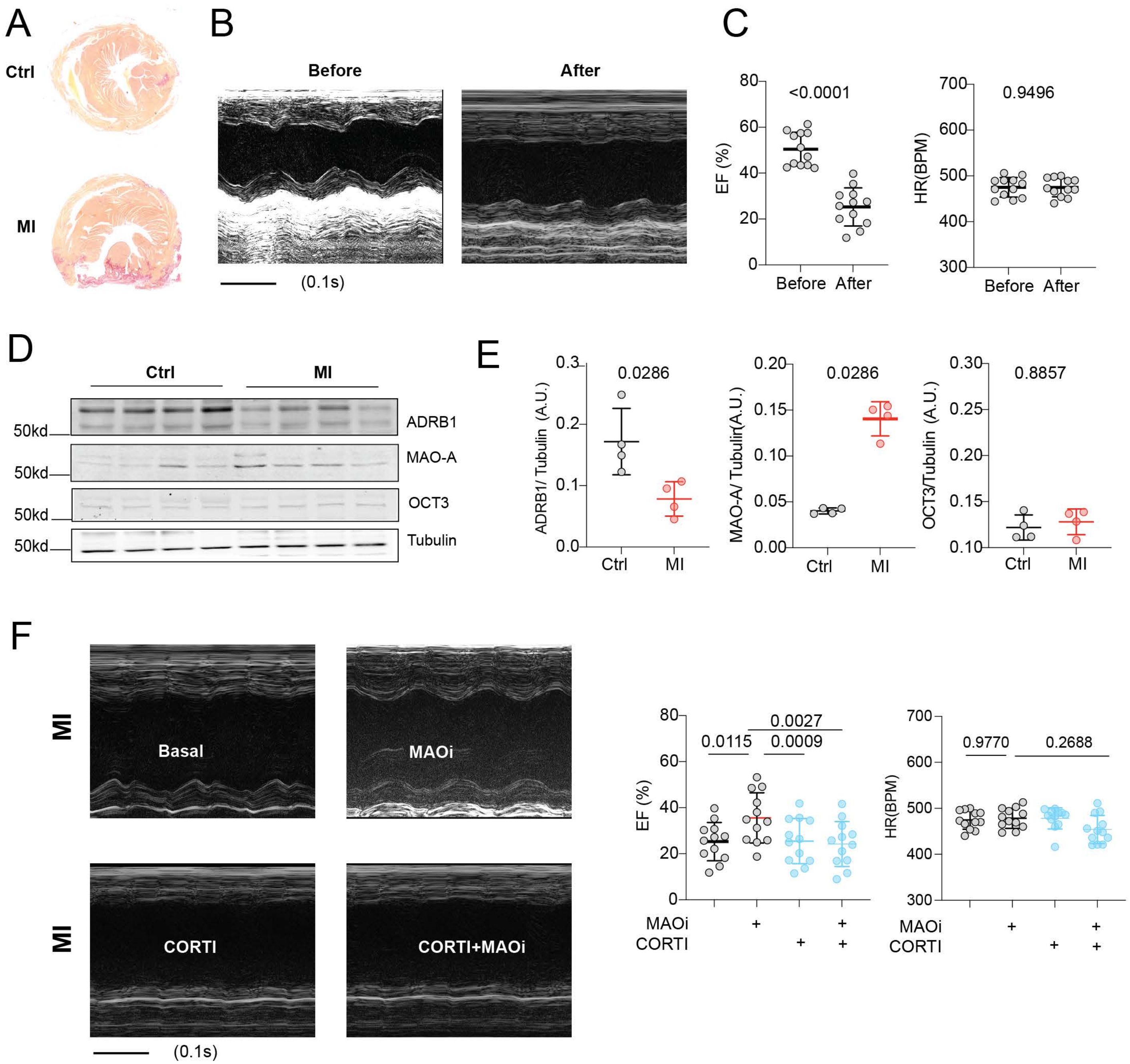

Supplement: Supplementary file 1 — (PDF 1754 KB) [file 395_2022_944_MOESM1_ESM.pdf]
