# Supplementary material for: Monoamine oxidase A and organic cation transporter 3 coordinate intracellular β1AR signaling to calibrate cardiac contractile function
Source: Basic Res Cardiol. 2022 Jul 17;117(1):37. doi: 10.1007/s00395-022-00944-5 (PMC9288959; doi:10.1007/s00395-022-00944-5)
Supplement: Supplementary file 2 — (DOCX 51 KB) [file 395_2022_944_MOESM2_ESM.docx]

**Online Table 1. Cardiac dose-dependent response of dobutamine injection in WT mice.** WT mice were injected with a serial dose of dobutamine (DOB, *i.p.*), sequentially. Cardiac function was recorded by M-mode echocardiography at baseline for 2 minutes and 8 minutes after each drug injection. Data shown are mean ± SD, n = 8. RM One-way ANOVA (RM One-way ANOVA) with Tukey’s multiple comparison test was performed and all pairwise comparison was applied. ^*^ p<0.05, ^**^ p<0.01, ^***^ p<0.001, ^****^ p<0.0001 compared with Baseline.

| Group |  | DOB (µg/kg) | | | |
| --- | --- | --- | --- | --- | --- |
|  | Baseline | 1 | 10 | 100 | 1000 |
| HR (bpm) | 467.6 ± 18.17 | 466.4 ± 20.62 | 450 ± 18.92 | 502.9 ± 22.55^**^ | 530.5 ± 32.38^***^ |
| IVS; d(mm) | 0.6266 ± 0.1316 | 0.6990 ± 0.1647 | 0.7128 ± 0.05778 | 0.7209 ± 0.04016 | 0.7532 ± 0.04027 |
| IVS; s(mm) | 0.6980 ± 0.04570 | 0.7603 ± 0.03051 | 0.7964 ± 0.03690^*^ | 0.9128 ± 0.0441^**^ | 0.9432 ± 0.05281^***^ |
| LVID; d(mm) | 3.192 ± 0.4618 | 3.154 ± 0.5115 | 2.991 ± 0.4728^*^ | 2.880 ± 0.4108^***^ | 2.807 ± 0.4352^****^ |
| LVID; s(mm) | 2.431 ± 0.3848 | 2.313 ± 0.4062 | 2.028 ± 0.3917^**^ | 1.624 ± 0.3440^***^ | 1.366 ± 0.4219^***^ |
| LVPW; d(mm) | 0.7620 ± 0.07936 | 0.7300 ± 0.07610 | 0.7329 ± 0.0914 | 0.7938 ± 0.09764 | 0.8412 ± 0.1017 |
| LVPW; s(mm) | 0.9911 ± 0.1464 | 1.115 ± 0.1112^*^ | 1.149 ± 0.1582 | 1.360 ± 0.1713^***^ | 1.565 ± 0.1871^***^ |
| EF % | 48.86 ± 5.400 | 53.46 ± 5.765^*^ | 61.88 ± 7.603^***^ | 75.55 ± 9.466^****^ | 83.24 ± 8.476^****^ |
| FS % | 23.90 ± 3.155 | 26.71 ± 3.623 | 32.30 ± 5.137^**^ | 43.67 ± 8.926^**^ | 51.87 ± 10.38^***^ |
| LV Mass (mg) | 66.65 ± 11.41 | 68.59 ± 14.23 | 63.64 ± 12.30 | 64.42 ± 11.86 | 66.65 ± 12.18 |
| LV Vol; d(μL) | 41.92 ± 15.21 | 41.02 ± 16.83 | 36.00 ± 14.87^*^ | 32.62 ± 11.85^**^ | 30.80 ± 12.27^***^ |
| LV Vol; s(μL) | 21.62 ± 9.028 | 19.26 ± 8.999 | 13.99 ± 7.910^**^ | 8.018 ± 4.077^**^ | 5.545 ± 4.316^***^ |

**Online Table 2. MAO-A inhibition enhanced cardiac contraction in WT mice.** In the absence (Ctrl) or presence of MAO-A inhibitor (MAOi, Clorgyine*, i.p.,* 0.4mg/kg), WT mice were treated with epinephrine (EPI, 100μg/kg, *i.p,* n = 9). M-mode echocardiography was recorded at baseline for 2 minutes and 8 minutes after each drug injection, maximal cardiac response was reported. Data shown are mean values ± SD. RM one-way ANOVA with Tukey’s multiple comparison correction was performed to analyze the differences. All pairwise comparison was applied * p<0.05, ** p<0.01 *** p<0.001, *** p<0.0001 compared to Baseline (Ctrl); ^&^ p<0.05, ^&&^ p<0.01, ^&&&^ p<0.001 compared to Baseline (MAOi); ^#^ p<0.05, ^##^ p<0.01, ^###^ p<0.001 compared with EPI (Ctrl).

| Group | Ctrl | | MAOi | |
| --- | --- | --- | --- | --- |
|  | Baseline | EPI | Baseline | EPI |
| HR (bpm) | 466.9 ± 37.70 | 514.2 ± 41.61 | 481.6 ± 33.89 | 555.9 ± 63.10^*, &^ |
| IVS; d(mm) | 0.7453 ± 0.1361 | 0.7867 ± 0.08429 | 0.7256 ± 0.07926 | 0.8591 ± 0.1948 |
| IVS; s(mm) | 1.050 ± 0.1493 | 1.200 ± 0.1175 | 1.038 ± 0.062 | 1.490 ± 0.2454^**, #, &&^ |
| LVID; d(mm) | 3.435 ± 0.3012 | 3.152 ± 0.3048^*^ | 3.374 ± 0.2663 | 2.875 ± 0.4756^*, &^ |
| LVID; s(mm) | 2.642 ± 0.2547 | 2.398 ± 0.2987^*^ | 1.967 ± 0.2530 | 1.240 ± 0.5536^&&&, #^ |
| LVPW; d(mm) | 0.7333 ± 0.1065 | 0.7442 ± 0.1077 | 0.7347 ± 0.1519 | 0.8275 ± 0.1086 |
| LVPW; s(mm) | 0.9890 ± 0.1295 | 1.237 ± 0.2636^*^ | 1.066 ± 0.1672 | 1.472 ± 0.2640^**, &^ |
| EF % | 47.50 ± 4.441 | 67.57 ± 6.059^***^ | 59.04 ± 6.711^**^ | 87.57 ± 8.90^&&, ###^ |
| FS % | 23.11 ± 2.713 | 37.61 ± 4.979^****^ | 29.09 ± 4.231 | 58.59 ± 12.84^&&&, ##^ |
| LV Mass (mg) | 82.38 ± 11.61 | 75.67 ± 14.62 | 78.76 ± 12.58 | 74.36 ± 12.55 |
| LV Vol; d(μL) | 49.14 ± 10.87 | 40.02 ± 9.912^*^ | 46.99 ± 9.393 | 32.81 ± 13.01^*, &^ |
| LV Vol; s(μL) | 25.96 ± 6.146 | 12.53 ± 4.376^****^ | 20.61 ± 6.846 | 5.019 ± 4.643^****, &&&,#^ |

**Online Table 3. MAO-A inhibition did not affect cardiac response to dobutamine.** In the absence (Ctrl) or presence of MAOi (*i.p.,* 0.4mg/kg), WT mice were treated with DOB (100μg/kg, *i.p.,* n = 9). M-mode echocardiography was recorded at baseline for 2 minutes and 8 minutes after each drug injection, maximal cardiac response was reported. Data shown are mean values ± SD. RM one-way ANOVA with Tukey’s multiple comparison correction was performed to analyze the differences. All pairwise comparison was applied. * p<0.05, ** p<0.01 *** p<0.001, *** p<0.0001 compared to Baseline (Ctrl); ^&&&^ p<0.001, ^&&&&^ p<0.0001 compared to Baseline (MAOi).

| Group | Ctrl | | MAOi | |
| --- | --- | --- | --- | --- |
|  | Baseline | DOB | Baseline | DOB |
| HR (bpm) | 448.1 ± 42.47 | 503.4 ± 34.03^*^ | 472.9 ± 31.46 | 523.4 ± 26.86^*, &&&^ |
| IVS; d(mm) | 0.6707 ± 0.2212 | 0.7339 ± 0.1575 | 0.6503 ± 0.1115 | 0.7419 ± 0.1276 |
| IVS; s(mm) | 0.8708 ± 0.2331 | 1.079 ± 0.2441 | 1.036 ± 0.1683 | 1.198 ± 0.1928^**^ |
| LVID; d(mm) | 3.485 ± 0.3568 | 3.045 ± 0.4294^*^ | 3.406 ± 0.4585 | 2.993 ± 0.4201^*, &&&&^ |
| LVID; s(mm) | 2.630 ± 0.2977 | 1.644 ± 0.4680^****^ | 2.508 ± 0.4612 | 1.645 ± 0.3090^***, &&&^ |
| LVPW; d(mm) | 0.6471 ± 0.03759 | 0.7603 ± 0.07401^*^ | 0.7627 ± 0.09451 | 0.8417 ± 0.1073^**^ |
| LVPW; s(mm) | 0.9318 ± 0.1211 | 1.355 ± 0.1572^**^ | 1.042 ± 0.1146 | 1.417 ± 0.09388^***, &&&^ |
| EF % | 49.8 ± 5.860 | 78.73 ± 8.851^***^ | 55.57 ± 5.482^*^ | 77.56 ± 5.927^****，&&&&^ |
| FS % | 24.54 ± 3.534 | 46.87 ± 8.568^****^ | 27.95 ± 3.717 | 45.13 ± 5.794^&&&, ##^ |
| LV Mass (mg) | 73.32 ± 22.92 | 69.16 ± 15.20 | 76.41 ± 12.35 | 72.62 ± 11.33 |
| LV Vol; d(μL) | 51.08 ± 12.04 | 37.34 ± 12.55^*^ | 48.12 ± 15.85 | 35.81 ± 12.51.^*, &&&^ |
| LV Vol; s(μL) | 25.80 ± 6.998 | 8.767 ± 6.105^***^ | 21.98 ± 8.878 | 8.175 ± 4.062^***, &&&^ |

**Online Table 4. Decynium-22 reduced inotropic response to epinephrine in WT mice.** WT mice were injected (*i.p.*) with EPI, Decynium-22 (D22) or cotreated with D22+EPI. D22 was pretreated for 5 minutes for drug coadministration. 5 minutes M-mode echocardiography was recorded at baseline for 2 minutes and 8 minutes after each drug injection, maximal cardiac response was reported EPI = 100 µg/kg, D22 = 200 µg/kg. Data shown are mean values ± SD, n= 9. RM one-way ANOVA followed by Tukey’s multiple comparison was performed to analyze the statistical significance of each group and every other group. All pairwise comparison was applied. ^*^ p<0.05, ^**^ p<0.01, ^***^ p<0.001, ^****^ p<0.0001 compared with Baseline; ^#^ p<0.05, ^##^ p<0.01compared with D22, ^$^ p<0.05, ^$$^ p<0.01, ^$$$^ p<0.01 compared with EPI.

| Group | WT mice (n = 9) | | | |
| --- | --- | --- | --- | --- |
|  | Baseline | EPI | D22 | D22+EPI |
| HR (bpm) | 470.9 ± 19.68 | 479.8 ± 25.20 | 401.8 ± 23.59^*^ | 403.2 ± 54.27^****, $$$^ |
| IVS; d(mm) | 0.6650 ± 0.05857 | 0.6511 ± 0.09007 | 0.6982 ± 0.08576 | 0.6985 ± 0.1088 |
| IVS; s(mm) | 0.8193 ± 0.08386 | 0.9420 ± 0.1739 | 0.8445 ± 0.08677 | 0.9008 ± 0.1295 |
| LVID; d(mm) | 3.193 ± 0.2667 | 3.074 ± 0.2492 | 3.361 ± 0.3201 | 3.204 ± 0.2867 |
| LVID; s(mm) | 2.481 ± 0.1833 | 1.903 ± 0.2634^***^ | 2.612 ± 0.2269 | 2.193 ± 0.2077^**, #, $$^ |
| LVPW; d(mm) | 0.7591 ± 0.1365 | 0.6905 ± 0.2275 | 0.7128 ± 0.1354 | 0.7035 ± 0.1094 |
| LVPW; s(mm) | 0.9572 ± 0.1589 | 1.099 ± 0.3177^****^ | 0.9556 ± 0.1283 | 1.067 ± 0.1435 |
| EF % | 46.07 ± 2.871 | 69.75 ± 5.934^****^ | 45.74 ± 5.378 | 60.55 ± 6.089^****,##,$^ |
| FS % | 22.22 ± 1.739 | 38.27 ± 4.755^****^ | 22.18 ± 3.150 | 31.48 ± 4.118^***,##, $^ |
| LV Mass (mg) | 70.03 ± 14.87 | 62.03 ± 22.76 | 75.10 ± 15.17 | 69.02 ± 14.99 |
| LV Vol; d(μL) | 41.14 ± 8.259 | 37.49 ± 7.621 | 46.73 ± 10.20 | 41.57 ± 8.993 |
| LV Vol; s(μL) | 22.08 ± 4.009 | 11.57 ± 4.065^***^ | 25.17 ± 5.082 | 16.31 ± 3.880^**,#, $$$^ |

**Online Table 5. Diminished inotropic cardiac response in β_1_-KO mice.** β_1_-KO mice were injected (*i.p.*) with 100 µg/kg EPI or 0.4 mg/kg MAOi. M-mode echocardiography was recorded at baseline for 2 minutes and 8 minutes after each drug injection, maximal cardiac response was reported Data shown are mean values ± SD, n= 8. RM one-way ANOVA followed by Tukey’s multiple comparison was performed to analyze the statistical significance of each group and every other group. All pairwise comparison was applied.

| Group | β_1_-KO (n = 8) | | |
| --- | --- | --- | --- |
|  | Baseline | EPI | MAOi |
| HR (bpm) | 387.8 ± 69.05 | 375.1 ± 59.10 | 400.3 ± 43 |
| IVS; d(mm) | 0.6411 ± 0.1185 | 0.6773 ± 0.1173 | 0.7324 ± 0.1908 |
| IVS; s(mm) | 0.8288 ± 0.1394 | 0.8446 ± 0.1639 | 0.9331 ± 0.2082 |
| LVID; d(mm) | 3.635 ± 0.2258 | 3.449 ± 0.2706 | 3.388 ± 0.4071 |
| LVID; s(mm) | 2.813 ± 0.1994 | 2.635 ± 0.2435 | 2.527 ± 0.3304 |
| LVPW; d(mm) | 0.6972 ± 0.1057 | 0.7580 ± 0.1403 | 0.7177 ± 0.1943 |
| LVPW; s(mm) | 0.9197 ± 0.1048 | 0.9438 ± 0.1641 | 0.9771 ± 0.1822 |
| EF % | 46.09 ± 5.864 | 47.67 ± 8.213 | 51.34 ± 3.236 |
| FS % | 22.56 ± 3.596 | 23.50 ± 5.222 | 25.48 ± 1.900 |
| LV Mass (mg) | 80.40 ± 21.73 | 80.12 ± 18.78 | 77.62 ± 17.49 |
| LV Vol; d(μL) | 55.99 ± 8.557 | 49.53 ± 8.823 | 47.98 ± 14.49 |
| LV Vol; s(μL) | 30.12 ± 5.361 | 25.77 ± 5.735 | 23.52 ± 8.000 |

**Online Table 6. β-adrenergic stimulation of cardiac function in MAO-A-FF vs CKO mice.** Mice were injected (*i.p.*) with 100 µg/kg DOB or EPI. M-mode echocardiography was recorded at baseline for 2 minutes and 8 minutes after each drug injection, maximal cardiac response was reported. Data shown are mean values ± SD, n= 9 FF, 9 CKO. All pairwise RM One-way ANOVA with Tukey’s multiple comparison test was performed to analyze. * p<0.05, ** p<0.01, *** p<0.001, **** p<0.0001 compared with FF Baseline; # p<0.05, ### p<0.001, #### p<0.0001 compared with CKO Baseline; $ p<0.05, $$ p<0.01 compared with FF EPI.

| Group | MAO-A-FF | | | MAO-A-CKO | | |
| --- | --- | --- | --- | --- | --- | --- |
|  | Baseline | EPI | DOB | Baseline | EPI | DOB |
| HR (bpm) | 466.6 ± 33.91 | 486.7 ± 25.51 | 557.2 ± 62.68^****^ | 411.4 ± 40.84 | 536.9 ± 57.73^###, $^ | 523.2 ± 54.85^##^ |
| IVS; d(mm) | 0.5953 ± 0.04177 | 0.6569 ± 0.1380 | 0.6375 ± 0.1092 | 0.6336 ± 0.05130 | 0.7755 ± 0.1498 | 0.6152 ± 0.1079 |
| IVS; s(mm) | 0.8646 ± 0.1214 | 1.060 ± 0.1881^****^ | 1.179 ± 0.1510^****^ | 0.9784 ± 0.07418 | 1.244± 0.09471^####, $$^ | 1.197 ± 0.1772^####^ |
| LVID; d(mm) | 3.611 ± 0.4397 | 3.393 ± 0.4433 | 3.141 ± 0.2457 | 3.669 ± 0.4542 | 3.020 ± 0.6203^#^ | 3.227 ± 0.2134 |
| LVID; s(mm) | 2.763 ± 0.3459 | 2.021 ± 0.3684^***^ | 1.600 ± 0.2019^****^ | 2.516 ± 0.3632 | 1.484 ± 0.5455^####, $^ | 1.652 ± 0.1759^####^ |
| LVPW; d(mm) | 0.6423 ± 0.09080 | 0.6845 ± 0.08599 | 0.7235 ± 0.07446 | 0.7516 ± 0.07076 | 0.8287 ± 0.1700 | 0.8910 ± 0.1188^$^ |
| LVPW; s(mm) | 0.9100 ± 0.09863 | 1.203 ± 0.1823^**^ | 1.326 ± 0.1752^****^ | 1.111 ± 0.1452 | 1.498 ± 0.2266^###, $$^ | 1.494 ± 0.1179^###^ |
| EF % | 47.57 ± 6.555 | 70.32 ± 5.723^****^ | 78.98 ± 3.760^****^ | 60.08 ± 5.963^***^ | 85.85 ± 6.383^####, $$^ | 83.22 ± 3.685^####^ |
| FS % | 23.43 ± 3.932 | 40.76 ± 4.650^****^ | 49.05± 5.477^****^ | 31.48 ± 4.143 | 52.64 ± 11.75^####, $$^ | 48.85 ± 3.891^####^ |
| LV Mass (mg) | 71.32 ± 16.46 | 70.86 ± 15.79 | 64.63 ± 17.96 | 85.02 ± 19.54 | 75.57 ± 20.73 | 76.94 ± 13.86 |
| LV Vol; d(μL) | 55.94 ± 15.64 | 48.33 ± 15.10 | 39.49 ± 7.466^**^ | 58.16 ± 16.54 | 37.72 ± 16.01^##^ | 42.07 ± 6.542^##^ |
| LV Vol; s(μL) | 29.26 ± 8.554 | 13.77 ± 5.839^****^ | 7.368 ± 2.104^****^ | 23.40 ± 7.952 | 7.236 ± 5.412^####^ | 7.936 ± 2.201^####^ |

**Online Table 7. Blocking OCT3 by corticosterone prevented the β-adrenergic stimulation of cardiac function in MAO-A-FF and CKO mice.** Mice were injected (*i.p.*) with EPI in the absence or presence of CORTI. CORTI was pretreated (*i.p.*) for 5 minutes for drug coadministration. M-mode echocardiography was recorded at baseline for 2 minutes and 8 minutes after each drug injection, and maximal cardiac response was reported. Data shown are mean values ± SD, n= 8 FF, 8 CKO. EPI = 100 µg/kg, CORTI = 200 µg/kg. One-way ANOVA with Tukey’s multiple comparison test was performed to analyze, all pairwise comparison was applied. ^*^ p<0.05, ^**^ p<0.01, ^***^ p<0.001, ^****^ p<0.0001 compared with Baseline (FF); ^##^p<0.01, ^###^ p<0.01, ^####^ p<0.0001 compared with Baseline (CKO); ^$^ p<0.05 compared with EPI (FF); ^&&^ p<0.01, ^&&&&^ p<0.0001 compared with EPI (CKO).

| Group | MAO-A-FF | | | | MAO-A-CKO | | | |
| --- | --- | --- | --- | --- | --- | --- | --- | --- |
|  | Baseline | EPI | CORTI | CORTI+EPI | Baseline | EPI | CORTI | CORTI+EPI |
| HR (bpm) | 470 ± 26.8 | 504 ± 14.1 | 463 ± 44.6 | 469 ± 44.3 | 464 ± 28.7 | 549 ± 37.0^####^ | 471 ± 33.4 | 450 ± 25.5^&&&&^ |
| IVS; d(mm) | 0.579± 0.0349 | 0.621 ± 0.0747 | 0.688 ± 0.108 | 0.736 ± 0.134 | 0.650 ± 0.0882 | 0.819 ± 0.156^$^ | 0.680 ± 0.126 | 0.710 ± 0.147 |
| IVS; s(mm) | 0.867 ± 0.00931 | 1.00 ± 0.116 | 0.943 ± 0.130 | 1.01 ± 0.209 | 0.972 ± 0.0819 | 1.21 ± 0.131 | 0.914 ± 0.159 | 1.01 ± 0.233 |
| LVID; d(mm) | 3.45 ± 0.396 | 3.33 ± 0.477 | 3.26 ± 0.286 | 3.09 ± 0.188 | 3.25 ± 0.227 | 2.82 ± 0.489 | 3.43 ± 0.434 | 3.28 ± 0.471 |
| LVID; s(mm) | 2.63 ± 0.353 | 2.03 ± 0.296^*^ | 2.48 ± 0.299 | 2.16 ± 0.225 | 2.22 ± 0.220 | 1.44 ± 0.460^##, $^ | 2.65 ± 0.374 | 2.28 ± 0.497^&&&^ |
| LVPW; d(mm) | 0.654 ± 0.0913 | 0.692 ± 0.0707 | 0.669 ± 0.192 | 0.755 ± 0.194 | 0.711 ± 0.0971 | 0.756 ± 0.129 | 0.644 ± 0.0964 | 0.640 ± 0.0804 |
| LVPW; s(mm) | 0.906 ± 0.101 | 1.18 ± 0.109^*^ | 0.905 ± 0.197 | 1.12 ± 0.243 | 1.07 ± 0.121 | 1.25 ± 0.153 | 0.861 ± 0.111 | 1.01 ± 0.171 |
| EF % | 48.4 ± 5.80 | 70.5 ± 3.72^****^ | 47.0 ± 5.69 | 56.1 ± 3.33^$$$^ | 61.1 ± 4.23^**^ | 81.8 ± 7.46^####, $^ | 47.0 ± 4.04 | 59.0 ± 11.7^&&&&^ |
| FS % | 23.8 ± 3.41 | 38.9 ± 3.22^***^ | 24.0 ± 4.05 | 30.3 ± 5.52^&&&&^ | 31.8 ± 2.94 | 50.6 ± 11.7^####, $$^ | 22.69 ± 2.30 | 30.9 ± 8.02.^&&&&^ |
| LV Mass (mg) | 66.2 ± 17.3 | 67.5 ± 17.6 | 60.4 ± 15.8 | 71.8 ± 21.7 | 67.5 ± 11.6 | 65.6 ± 15.3 | 70.6 ± 14.7 | 68.3 ± 17.5 |
| LV Vol; d(μL) | 50.0 ± 13.9 | 46.4 ± 16.4 | 43.0 ± 8.79 | 38.0 ± 5.54 | 42.9 ± 7.20 | 31.2 ± 10.1 | 49.5 ± 15.0 | 44.7 ± 14.8 |
| LV Vol; s(μL) | 26.1 ± 8.69 | 13.7 ± 4.92^*^ | 22.2 ± 6.01 | 15.7 ± 3.91 | 16.9 ± 4.25 | 6.31 ± 2.98^#^ | 26.5 ± 9.38 | 19.1 ± 9.57^&&^ |

**Online Table 8. OCT3 deletion abrogated impact of MAOi on β-adrenergic stimulation of cardiac function.** WT and OCT3-KO mice were injected (*i.p.*) with EPI in the absence or presence of MAOi (*i.p.*). M-mode echocardiography was recorded at baseline for 2 minutes and 8 minutes after each drug injection, maximal cardiac response was reported. Data shown are mean ± SD, n= 8 WT, 8 OCT3-KO. MAOi = 0.4 mg/kg, EPI = 100 µg/kg, CORTI = 200 µg/kg. One-way ANOVA with Tukey’s multiple comparison test was performed to analyze, all pairwise comparison was applied. ^*^ p<0.05, ^**^ p<0.01, ^***^ p<0.001, ^****^ p<0.0001 compared with Basaline (WT), ^#^ p<0.05 compared with Basaline (OCT3-KO), ^$^ p<0.05, ^$$^ p<0.01, ^$$$^ p<0.01 compared with EPI (WT), ^&&^ p<0.01, ^&&&^ p<0.001, ^&&&&^ p<0.0001 compared with MAOi+EPI (WT).

| Group | WT | | | | OCT3KO | | | |
| --- | --- | --- | --- | --- | --- | --- | --- | --- |
|  | Baseline | EPI | MAOi | MAOi+EPI | Baseline | EPI | MAOi | MAOi+EPi |
| HR (bpm) | 470.6 ± 8.932 | 500.3 ± 22.68 | 480.4 ± 18.11 | 550.4 ± 33.15^&^ | 499.1 ± 40.06 | 474.0 ± 24.61 | 460.1 ± 36.32 | 472.0 ± 31.03^&&&&^ |
| IVS; d(mm) | 0.7080± 0.09892 | 0.7815 ± 0.07318 | 0.6670 ± 0.1056 | 0. 7539± 0.1059 | 0.7879 ± 0.1315 | 0.812 ± 0.1546 | 0.7153 ± 0.07493 | 0.7057 ± 0.09266 |
| IVS; s(mm) | 0.968 ± 0.0977 | 1.08 ± 0.116 | 1.02 ± 0.129 | 1.18 ± 0.114 | 1.14 ± 0.00971 | 1.14 ± 0.207 | 0.974 ± 0.118 | 1.14 ± 0.154 |
| LVID; d(mm) | 2.98 ± 0.339 | 2.63 ± 0.433 | 2.95 ± 0.263 | 2.52 ± 0.282 | 3.93 ± 0.539^***^ | 3.88 ± 0.579 | 3.92 ± 0.459 | 3.74 ± 0.358^&&&&^ |
| LVID; s(mm) | 2.30 ± 0.254 | 1.70 ± 0.334^*^ | 2.02 ± 0.234 | 1.30 ± 0.227 | 3.10 ± 0.435^**^ | 2.85 ± 0.567 | 3.15 ± 0.472 | 2.67 ± 0.331^&&&&^ |
| LVPW; d(mm) | 0.712 ± 0.0865 | 0.764 ± 0.111 | 0.706 ± 0.0997 | 0.756 ± 0.167 | 0.741 ± 0.0931 | 0.772 ± 0.119 | 0.788 ± 0.184 | 0.794 ± 0.193 |
| LVPW; s(mm) | 0.953 ± 0.0932 | 1.11 ± 0.0943 | 1.01 ± 0.186 | 1.27 ± 0.224 | 0.993 ± 0.128 | 1. 10± 0.143 | 0.995 ± 0.215 | 1.13 ± 0.176 |
| EF % | 47.25 ± 3.658 | 66.89 ± 5.069^****^ | 61.39 ± 4.246^***^ | 81.70 ± 4.474^&&&^ | 42.87 ± 7.419 | 53.07 ± 6.355^#,$^ | 41.98 ± 7.235 | 56.76 ± 7.773^&&&^ |
| FS % | 22.8 ± 2.17 | 35.5 ± 3.64^****^ | 31.8 ± 2.78^***^ | 48.7 ± 4.75^&&&&^ | 21.1 ± 1.70 | 26.9 ± 3.74^$$^ | 19.8 ± 5.34 | 28.7 ± 1.89^&&&&^ |
| LV Mass (mg) | 62.7 ± 14.7 | 58.1 ± 13.5 | 58.4 ± 12.0 | 52.5 ± 11.0 | 110 ± 33.9^**^ | 111 ± 28.7^$$$^ | 105 ± 25.9 | 98.3 ± 28.2^&&^ |
| LV Vol; d(μL) | 35.2 ± 9.43 | 26.4 ± 11.0 | 34.0 ± 7.46 | 23.1 ± 6.25 | 69.0 ± 24.1^**^ | 66.9 ± 25.3^$$$^ | 67.8 ± 18.8 | 60.4 ± 13.6^&&&^ |
| LV Vol; s(μL) | 18.50 ± 4.980 | 8.971 ± 4.951 | 13.27 ± 3.943 | 4.354 ± 2.058 | 39.13 ± 14.21^**^ | 32.63 ± 17.13^$$$^ | 40.58 ± 15.10 | 26.86 ± 7.838^&&^ |

**Online Table 9. Impaired cardiac response in ischemic heart failure mice.** WT mice were subjected to coronary artery ligation induced myocardial infarction (MI). Cardiac function of the mouse before and 3-days after MI were obtained by M-mode echocardiography. Data shown are mean values ± SD, n= 12. Paired *t* test was performed to analyze the statistical significance between before and after MI. ^*^ p<0.05, ^**^ p<0.01, ^****^ p<0.0001compared with Before MI.

| Group | WT (n = 12) | |
| --- | --- | --- |
|  | Before | After (MI) |
| HR (bpm) | 475.2 ± 21.46 | 474.7 ± 20.09 |
| IVS; d(mm) | 0.6954 ± 0.1095 | 0.7038 ± 0.1699 |
| IVS; s(mm) | 0.9219 ± 0.1323 | 0.7908 ± 0.2331 |
| LVID; d(mm) | 3.380 ± 0.3004 | 3.642 ± 0.5060 |
| LVID; s(mm) | 2.543 ± 0.3574 | 3.238 ± 0.5601^**^ |
| LVPW; d(mm) | 0.6633 ± 0.09175 | 0.8018 ± 0.1804^*^ |
| LVPW; s(mm) | 0.8935 ± 0.1105 | 0.9093 ± 0.1964 |
| EF % | 50.45 ± 7.378 | 25.29 ± 8.294^****^ |
| FS % | 25.06 ± 4.385 | 11.46 ± 3.959^****^ |
| LV Mass (mg) | 73.07 ± 20.86 | 95.26 ± 32.59^**^ |
| LV Vol; d(μL) | 47.31 ± 10.15 | 57.53 ± 19.84 |
| LV Vol; s(μL) | 24.01 ± 8.225 | 44.01 ± 18.72^**^ |

**Online Table 10. MAOi enhanced cardiac function in ischemic heart failure mice.** WT mice were injected (*i.p.*) with MAOi, CORTI or cotreated with CORTI+MAOi. CORTI was pretreated for 5 minutes for co-administration. M-mode echocardiography was recorded at baseline for 2 minutes and 8 minutes after each drug injection, maximal cardiac response was reported. MAOi =400 µg/kg, and CORTI = 200 µg/kg. Data shown are mean values ± SD, n= 12. All pairwise RM One-way ANOVA with Tukey’s multiple comparison correction was performed to analyze the statistical significance between different groups. ^*^ p<0.05, ^**^ p<0.01, compared with Baseline; # p<0.05, ### p<0.001, compared with MAOi.

| Group | MI mice (n = 12) | | | |
| --- | --- | --- | --- | --- |
|  | Baseline | MAOi | CORTI | CORTI+MAOi |
| HR (bpm) | 474.7 ± 20.09 | 478.3 ± 21.68 | 478.2 ± 22.93 | 454.0 ± 30.48^*^ |
| IVS; d(mm) | 0.7038 ± 0.1699 | 0.6775 ± 0.1797 | 0.5426 ± 0.1141 | 0.5832 ± 0.2122^**^ |
| IVS; s(mm) | 0.7908 ± 0.2331 | 0.8197 ± 0.2218^**^ | 0.6180 ± 0.1336 | 0.6622 ± 0.2555^**, #^ |
| LVID; d(mm) | 3.642 ± 0.5060 | 3.812 ± 0.4851 | 3.946 ± 0.7187 | 3.924 ± 0.5742^*^ |
| LVID; s(mm) | 3.238 ± 0.5601 | 3.201 ± 0.5724 | 3.509 ± 0.7884 | 3.506 ± 0.6416^#^ |
| LVPW; d(mm) | 0.8018 ± 0.1804 | 0.7939 ± 0.1908 | 0.7328 ± 0.2256 | 0.8250 ± 0.3331 |
| LVPW; s(mm) | 0.9093 ± 0.1964 | 1.021 ± 0.2357^****^ | 0.8261 ± 0.2449 | 0.9209 ± 0.3361 |
| EF % | 25.29 ± 8.294 | 35.60 ± 11.06^*^ | 25.51 ± 9.810^##^ | 24.26 ± 9.733^##^ |
| FS % | 11.46 ± 3.959 | 16.44 ± 5.846^*^ | 11.67 ± 4.709^##^ | 11.07 ± 4.695^#^ |
| LV Mass (mg) | 95.26 ± 32.59 | 102.0 ± 44.02 | 86.88 ± 32.27 | 103.2 ± 56.30 |
| LV Vol; d(μL) | 57.53 ± 19.84 | 63.85 ± 19.58 | 70.91 ± 30.98^*^ | 68.89 ± 24.17 |
| LV Vol; s(μL) | 44.01 ± 18.72 | 42.94 ± 18.43 | 54.91 ± 29.46 | 53.51 ± 23.43^#^ |
