# Supplementary material for: Monoamine oxidase A and organic cation transporter 3 coordinate intracellular β1AR signaling to calibrate cardiac contractile function
Source: Basic Res Cardiol. 2022 Jul 17;117(1):37. doi: 10.1007/s00395-022-00944-5 (PMC9288959; doi:10.1007/s00395-022-00944-5)
Supplement: Supplementary file 3 — (DOCX 59 KB) [file 395_2022_944_MOESM3_ESM.docx]

**Supplemental Information**

**ONLINE METHODS**

**Animals**

Male and Female 2–4-month-old mice and New Zealand White rabbits (3-6 months old) were used in this study. C57BL/6J WT, β_1_AR-KO, and OCT3-KO were described previously [5,7]. MAO-A-flox mice were gifted by Dr. Jean Shih at the University of Southern California and crossbred with MHC-CRE+ (Jackson Laboratory, Stock # 9704) to generate MAO-A flox/MHC-CRE+ (cardiac-specific knockout, CKO). Mice genotypes were confirmed by using PCR. All the experiment procedures, including tissue collection, echocardiogram, and myocardial infarction (MI) surgery were approved by the Institutional Animal Care and Use Committees (IACUC, Protocol 20234 and 20597) of the University of California at Davis and followed NIH and ARRIVE guidelines. All the animals were housed in an animal facility with controlled humidity (30%-70%), temperature (22˚C), lightning (12/12-hour cycle); and the animal brand information can be found in the Animal Resource Table. Mice were anesthetized with isoflurane (1-2%) in oxygen through a nose cone during echocardiography. Mice were humanely euthanized for tissue harvest and cell isolation under anesthesia with isoflurane (3%). The hearts were quickly excised and snap-frozen in liquid nitrogen and then transferred to a -80˚C freezer for long-term storage. Alternatively, hearts were quickly rinsed in a chilled buffer for cannulation to isolate myocytes.

**Chemical Reagents**

(-)-epinephrine bitartrate salt (L-3,4-Dihydroxy-α-(methylaminomethyl)benzyl alcohol D-hydrogen bitartrate salt, C_9_H_13_NO_3_ · C_4_H_6_O_6_, E4375), dobutamine hydrochloride ((±)-3,4-Dihydroxy-N-[3-(4-hydroxyphenyl)-1-methylpropyl]-β-phenethylamine hydrochloride, C_18_H_23_NO_3_·HCl, D0676), and L-(-)-norepinephrine (+)-bitartrate salt monohydrate (L-(−)-Noradrenaline (+)-bitartrate salt monohydrate, C_8_H_11_NO_3_·C_4_H_6_O_6_·H_2_O, A9512) from Sigma-Aldrich (I6504, St. Louis, MO) were freshly prepared to working concentration each day. Corticosterone (11β,21-Dihydroxy-4-pregnene-3,20-dione, C_21_H_30_O_4_, 27840), N-Methyl-N-propargyl-3-(2,4-dichlorophenoxy), and clorgyline (propylamine hydrochloride, C_13_H_15_Cl_2_NO·HCl, M3778) from Sigma-Aldrich were dissolved in DMSO and diluted to working concentration before use. Fluro-4 (N-[4-[6-[(acetyloxy)methoxy]-2,7-difluoro-3-oxo-3H-xanthen-9-yl]-2-[2-[2-[bis[2-[(acetyloxy)methoxy]-2 oxoethyl]amino]-5-methylphenoxy]ethoxy]phenyl]-N-[2-[(acetyloxy)methoxy]-2-oxoethyl]-, (acetyloxy)methyl ester 273221-67-3, C_51_H_50_F_2_N_2_O_23_, F14201, Life Technologies Corporation) was prepared in pluronic F-127 (P3000MP, Life Technologies Corporation). Decynium 22 (D22, 1-Ethyl-2-[(1-ethyl-2(1H)-quinolinylidene)methyl]quinolinium iodide) from Tocris Bioscience was dissolved in DMSO.

**Norepinephrine Measurement**

Norepinephrine concentrations in plasma, heart, and brain tissues were measured by a norepinephrine ELISA kit (KA1891, Abnova) at room temperature (22˚C) as described previously [5]. Heart and brain tissues (1 g/mL) were firstly homogenized in 9 ml cold PBS with 1 mmol/L protease inhibitor PMSF. Wash buffer and enzyme solution were prepared according to the manufacturer's instructions. 300 µL of samples and 10 µL of standards were added to an extraction plate and mixed with 250 µL water, 50 µL assay buffer, and 50 µL extraction buffer. The samples were then incubated and shaked for 30 minutes. The plate was then washed twice with 1 mL wash buffer before adding 150 µL acylation and 25 µL acylation reagent and incubating for 15 minutes. The plate was washed before adding 150 µL hydrochloric acid and incubating 10 minutes. After the above extraction, 20 µL of the samples and standards were incubated with the enzyme solution for 30 minutes in the Noradrenaline Microtiter Strips. 50 µL of the noradrenaline antiserum was added to the strips and incubated for 2 hours. The supernatant solutions were removed before washing the plate 3 times. 100 µL conjugated antibody was pipetted into the plate and incubated for 30 minutes. After rinsing the strips with wash buffer, 100 µL substrate was added to each well of the strips and incubated for 25 minutes. The reaction was stopped by a 100 µL stop solution. The OD value of each well was measured at 450nm using a SpectraMax M5 plate reader (Molecular Devices, Sunnyvale, CA).

**Tissue Harvesting and Plasma Collection**

Before sacrifice, mice were under anesthesia with 5% isoflurane in oxygen for 3 minutes. The heart and brain tissues were quickly excised and put into liquid nitrogen after thoracotomy. Mice body weight and organ weight were recorded. All the tissues were stored at -80ºC before biochemistry, ROS, and ELISA assay. Whole blood was collected in heparin containing tubes and then centrifuged at 1000 x g at 4ºC for 30 minutes. The supernatant plasma was collected and stored at -80 ºC before the ELISA assay.

**Adult Rabbit Ventricular Myocyte Isolation**

Rabbit adult ventricular myocytes (AVMs) were isolated as previously described [1]. Rabbits were anesthetized (induction with propofol 2 mg/kg followed by 2-5 % isoflurane in 100 % oxygen) and then sacrificed. After thoracotomy, the heart was quickly excised and rinsed in cold Ca^2+^-free minimum essential medium (MEM). The left coronary ostium was cannulated using a 4F catheter. After perfusion was established, the right atrium and non-perfused right ventricle free wall were removed, and the catheter was secured with a purse-string suture. The remainder of the isolation procedure was executed as previously described [1].

**Adult Mouse Ventricular Myocyte Isolation**

Male mice (2-4-month-old) were *i.p* injected with 5000 unites/kg heparin before anesthesia (3-4% Isoflurane). The heart was excised and perfused with the Langendorff perfusion system (ADInstruments) as previously described [7]. The heart was digested with 12.5 mg of collagenase II and 2.5 mg of protease XIV. The atrial was discarded, and the ventricle was dissociated with forceps. AVMs were centrifuged and pelleted at 500 rpm for 1 minute. Then the pellet was resuspended and recovered in perfusion buffer with a gradient of Ca^2+^ to 1 mmol/L.

**Cell Culture**

Freshly isolated rabbit and mouse AVMs were seeded on laminin (Life Technologies, Grand Island, NY)-coated 12-well plates. AVMs were seeded in MEM (M1018, Sigma-Aldrich, St. Louis, MO) with additional 4 mmol/L NaHCO_3_, 10 mmol/L HEPES, 1% PSG, 0.2% BSA, 6.25 µmol/L blebbistatin (#13013, Cayman Chemical), and 10% FBS as previously reported [3]. The medium was gently replaced by serum-free MEM (4 mmol/L NaHCO_3_, 10 mmol/L HEPES, 1% PSG, 0.2% BSA, and 6.25 µmol/L blebbistatin) after 2 hours of seeding. MAOi (5 µmol/L) and CORTI (2 µmol/L) were used to pretreat AVMs for 5 minutes followed by 5-minutes incubation of β-agonists (1 µmol/L dobutamine or 1 µmol/L epinephrine). Rabbit AVMs were infected with or without MAO-A overexpression adenovirus for 24 hours before β-agonists stimulation (norepinephrine, epinephrine, and dobutamine; 5 minutes). AVMs were harvested for western blotting.

**Adenovirus Construction and Infection**

A flag-tagged MAO-A cytoplasmic domain (aa1-aa497) was cloned by PCR amplification with a forward primer containing EcoRI enzymatic digestion site and a reverse primer containing XhoI enzymatic digestion site. The amplified product was inserted into the EcoRI/XhoI sites of a pcDNA3.1-mCherry plasmid to produce flag-MAO-A-cyto-mCherry plasmid. The recombinant adenovirus encoding flag-MAO-A-cyto-mCherry was then generated with the pAdEasy system (Qbiogene, Carlsbad, CA). Freshly isolated AVMs were plated on mouse laminin (Life Technologies, Grand Island, NY) coated dishes with culture mediums. AVMs were infected with adenovirus for 24 hours for biochemistry or 36 hours for FRET assay. The brick-shape and t-tubule structures were still maintained in AVMs during culture [3].

**Western Blotting**

Left ventricle tissues and AVMs were lysed in the buffer (25 mmol/L Hepes, pH 7.4; 5 mmol/L EDTA; 150 mmol/L NaCl; 0.5 % Triton X-100, 2 mmol/L Na_3_VO_4_, 1 mmol/L PMSF, 10 mmol/L NaF, 5 mmol/L Bestatin, and 2 µg/mL Pepstain A). Equal amounts of protein were mixed with 2 x SDS loading buffer (#161-0737, Bio-Rad) and then resolved on SDS-PAGE gels. Proteins were detected with anti-phospho-PLB serine 16 (1:1000, A010-12, Badrilla, Leeds, United Kingdom), anti-PLB (1:1000; A010-14, Badrilla, Leeds, United Kingdom), anti-phospho-RyR serine 2808 (1:1000, ab59225, Abcam), anti-RyR2 (1:500, MA3925, Thermo Fisher Scientific), anti-phospho-Troponin serine 23/24 (1:500, #4004, Cell Signaling Technology), anti-Troponin (1:500, #4002, Cell Signaling Technology), anti-phospho-Cav1.2 serine 1928 (1:1000, custom antibody from Abmart, NJ, USA), anti-Cav1.2 (1:500, #75-257, Antibodies Inc), anti-gamma-Tubulin (1:1000, T6567, Sigma-Aldrich), anti-MAO-A (H-70) (1:500, sc-20156, Santa Cruz Technology), anti-SERCA2 (1:1000; MAB2636, Millipore). All primary antibodies were incubated overnight at 4ºC and then revealed with IRDye 800 CW secondary antibodies (1:10000, #926-32210, Goat-anti-mouse or #926-32211 Goat-anti-rabbit, LI-COR) using Bio-Rad ChemiDoc MP Imagers (Bio-Rad Laboratories, Hercules, CA). The fluorescence density of individual bands was quantified using NIH Image J software (https://imagej.nih.gov/ij/).

**Fluorescent Resonance Energy Transfer (FRET) Assay**

As previously reported, a series of subcellular-anchored FRET biosensors (AKAR3, A-kinase activity reporter 3) were applied for the localized PKA activities [3, 5]. PM-AKAR3, the plasma membrane-anchored AKAR3, was generated by fusing with the sequence KKKKKKSKTKCVIM of K-Ras. SR-AKAR3, sarcoplasmic reticulum anchored AKAR3, was fused with the transmembrane domain of phospholamban [3, 5]. The recombinant adenoviruses were applied to express the FRET biosensors in AVMs. Thirty-six hours after infection, FRET images were recorded at room temperature (22˚C) on a Zeiss AX10microscope with a 40x/1.3 oil-immersion objective lens (Oberkochen, Germany). FRET image acquisition and intensity ratio measurement (YFP/CFP) were conducted through Metafluor software (Molecular Devices, Sunnyvale, CA). Briefly, CFP was excited at 430-455 nm, and emission fluorescence was collected at two wavelengths (475 nm for CFP and 535nm for YFP, 200 ms exposure) every 20 seconds, for 400-500 seconds (100 seconds at the baselines and 300-400 seconds post agonist stimulation). Image intensities were subtracted from the background, and the Δ YFP/CFP ratio was calculated and normalized to the baseline ratio (before drug stimulation). The maximal ΔYFP/CFP ratios were plotted on the bar graphs.

**ROS Measurement**

Oxidative stress (ROS) was determined by MDA formation measurement as previously reported [2]. MDA, an end-product of lipid peroxidation, was determined spectrophotometrically by quantifying 2-thiobarbituric acid reactive substances (TBARS).

**Echocardiography**

Cardiac function was measured using a Vevo 2100 imaging system with (VisualSonics) with a 22- to 55-MHz MS550D transducer. As previously described, a short-axis M model was applied to mice under anesthesia (2% isoflurane for induction and 1.5% for maintenance) [4]. The body temperature was maintained at 37˚C, and the respiratory rate, electrocardiogram (ECG), and heart rate were monitored. Cardiac function was recorded at the baselines and after intraperitoneal injection of clorgyline (MAOi, 0.4mg/kg), epinephrine (EPI, 100 µg/kg), or dobutamine (DOB, 1 µg/kg, 10 µg/kg, 100 µg/kg, or 1000 µg/kg) in the presence or absence of inhibitor corticosterone (CORTI, 200 µg/kg, *i.p.,* pretreated for 5 minutes). Cardiac function was recorded for 2 minutes at the baselines and for 8 minutes after each drug administration until it reached a steady stage. Maximal ejection fraction response was analyzed and reported.

**Acute MI**

8 weeks old male WT mice were subjected to the ligation of left anterior descending coronary artery (LAD) surgery as previously described [6]. Mice were pre-anesthetized with isoflurane (5.0%, 5 minutes, inhalation) and maintained with isoflurane (1.0-5.0%, inhalation) in oxygen flow to 0.8 L/minute. The trachea was intubated with a 22-G intravenous catheter over a 38-mm stiff, straight, tip-blunted needle and mechanically ventilated with supplemental oxygen at a respiratory rate of 110-130 breaths/min and tidal volume of 0.2 ml. Mice ECG was monitored with Bio Amps (AD instrument). The chest cavity was opened from the left sternal border in the 4th intercostal space (1.0-2.0cm length incision), and then the chest retractor was inserted to visualize the heart. The left anterior descending (LAD) coronary artery was identified under a surgical microscope and then ligated with an 8-0 nylon suture [ETHICON, Johnson&Johnson], inducing 30-40% infarction of the left ventricle. Infarction was confirmed by a change of ECG. The retractor was removed before closing the chest cavity with a 6-0 nylon suture. Analgesia was given after the surgery using 0.1 mg/kg of buprenorphine and twice daily for 48 hours of coverage.

**Contractility and Ca^2+^ Measurement**

Freshly isolated mouse AVMs were incubated with Fluo-4 AM (5 µmol/L, F14201, Invitrogen) for 15 minutes for Ca^2+^ measurement at room temperature 22˚C. AVMs were washed with Fluo-4-free buffer to remove nonspecifically associated dye and incubated for 10 minutes before being placed in beating buffer (NaCl 120 mmol/L, KCl 5.4 mmol/L, NaH2PO4 1.2 mmol/L, MgSO4 1.2 mmol/L, HEPES 20 mmol/L, Glucose 5.5 mmol/L, CaCl2 1mmol/L, pH 7.1). AVMs were pretreated with or without MAO-A inhibitor (5 µmol/L) or OCT3 inhibitor (2 µmol/L) for 5 minutes. Cells were then paced at 1 Hz with the electrical pacer (Grass Technology, Warwick, RI). Intracellular Ca^2+^ and cell length were recorded before (2 minutes) and after (6 minutes) drug stimulation with a Zeiss AX10 inverted microscope using 20 x objective at a 40 frames/second speed. Ca^2+^ transients and cell length responses reached steady states after 3-4 minutes of drug administration. The maximal Ca^2+^ and cell length after agonist treatment were analyzed and reported. Sarcomere shortening (SS%) was calculated with MetaMorph® software (Molecular Devices, Sunnyvale, CA). Ca^2+^ analysis was performed using GaiLab (custom-written software) as previously described [4, 5].

**Animal Resource Table**

| **Sex** | **Species** | **Vendor or Source** | **Background Strain** | **Other Information** | **Persistent ID / URL** |
| --- | --- | --- | --- | --- | --- |
| Male and Female | mouse | Johannes W. Hell and Paul J.Gasser, Marquette University | C57/BL6J | OCT3-KO | https://www.marquette.edu/biomedical-sciences/paul-gasser-research.php |
| Male and Female | mouse | Kevin Xiang, UCDavis | C57/BL6J | β_1_ARKO | https://health.ucdavis.edu/team/pharmacology/21783/yang-xiang---pharmacology-davis#.Xybd5y05SCM |
| Male and Female | mouse | Kevin Xiang UCDavis (self-breeded) | C57/BL6J | WT | https://health.ucdavis.edu/team/pharmacology/21783/yang-xiang---pharmacology-davis#.Xybd5y05SCM |
| Male and Female | mouse | Jean C. Shih, University of Southern California and Kevin Xiang UCDavis | C57/BL6J | MAO-A-CKO | https://health.ucdavis.edu/team/pharmacology/21783/yang-xiang---pharmacology-davis#.Xybd5y05SCM |
| Male | Rabbit | Donald M. Bers, UC Davis | New Zealand white rabbit |  | https://biology.ucdavis.edu/people/donald-bers |

**Reference**

1. Barbagallo F, Xu B, Reddy GR, West T, Wang Q, Fu Q, Li M, Shi Q, Ginsburg KS, Ferrier W, Isidori AM, Naro F, Patel HH, Bossuyt J, Bers D, Xiang YK (2016) Genetically Encoded Biosensors Reveal PKA Hyperphosphorylation on the Myofilaments in Rabbit Heart Failure. Circ Res 119:931–943. doi: 10.1161/CIRCRESAHA.116.308964

2. Kaludercic N, Takimoto E, Nagayama T, Feng N, Lai EW, Bedja D, Chen K, Gabrielson KL, Blakely RD, Shih JC, Pacak K, Kass DA, Lisa FD, Paolocci N (2010) Monoamine oxidase A mediated enhanced catabolism of norepinephrine contributes to adverse remodeling and pump failure in hearts with pressure overload. Circ Res 106:193–202. doi: 10.1161/CIRCRESAHA.109.198366

3. Reddy GR, West TM, Jian Z, Jaradeh M, Shi Q, Wang Y, Chen-Izu Y, Xiang YK (2018) Illuminating cell signaling with genetically encoded FRET biosensors in adult mouse cardiomyocytes. J Gen Physiol 150:1567–1582. doi: 10.1085/jgp.201812119

4. Wang Q, Wang Y, West TM, Liu Y, Reddy GR, Barbagallo F, Xu B, Shi Q, Deng B, Wei W, Xiang YK (2020) Carvedilol induces biased β1 adrenergic receptor-Nitric oxide synthase 3-cyclic guanylyl monophosphate signaling to promote cardiac contractility. Cardiovasc Res. doi: 10.1093/cvr/cvaa266

5. Wang Y, Shi Q, Li M, Zhao M, Reddy Gopireddy R, Teoh J-P, Xu B, Zhu C, Ireton KE, Srinivasan S, Chen S, Gasser PJ, Bossuyt J, Hell JW, Bers DM, Xiang YK (2021) Intracellular β1-Adrenergic Receptors and Organic Cation Transporter 3 Mediate Phospholamban Phosphorylation to Enhance Cardiac Contractility. Circ Res 128:246–261. doi: 10.1161/CIRCRESAHA.120.317452

6. Wang Y, Zhang Q, Zhong L, Lin M, Luo X, Liu S, Xu P, Liu X, Zhu YZ (2017) Apoptotic Protease Activating Factor-1 Inhibitor Mitigates Myocardial Ischemia Injury via Disturbing Procaspase-9 Recruitment by Apaf-1. Oxid Med Cell Longev 2017:9747296. doi: 10.1155/2017/9747296

7. Xu Bing, Li Minghui, Wang Ying, Zhao Meimi, Morotti Stefano, Shi Qian, Wang Qingtong, Barbagallo Federica, Teoh Jian-Peng, Reddy Gopireddy R., Bayne Elizabeth F., Liu Yongming, Shen Ao, Puglisi Jose L., Ge Ying, Li Ji, Grandi Eleonora, Nieves-Cintron Madeline, Xiang Yang K. (2020) GRK5 Controls SAP97-Dependent Cardiotoxic β1 Adrenergic Receptor-CaMKII Signaling in Heart Failure. Circ Res 127:796–810. doi: 10.1161/CIRCRESAHA.119.316319

**Online Figures**

**Online Figure I. Inotropic response in WT mice.** Cardiac function was recorded for 2 minutes at baseline and for 8 minutes after each dose injection. The maximal cardiac response was reported, (**A, B**) Dose-dependent response of DOB-promoted EF (A) and HR (B) in WT mice. Mice were sequentially *i.p.* injected with 1, 10, 100, and 1000 µg/kg of DOB. n = 8. (**C, D**) WT mice were injected with 100 µg/kg EPI or DOB in the absence or the presence of MAOi pretreatment (0.4 mg/kg, *i.p.,* 5 minutes). Cardiac EF were recorded and analyzed. ΔEF = ∆EF_EPI_ (EF_MAOi+EPI_ -EF_MAOi_ vs EF_EPI_ -EF_baseline_) or ∆EF_DOB_ (EF_MAOi+DOB_ -EF_MAOi_ vs EF_DOB_ -EF_baseline_). n = 9. Data are shown as mean ± SD. RM One-Way ANOVA was performed for panels (A, B) and followed by Tukey’s multiple comparison correction. Two-tailed paired *t*-test was applied for the panels (C, D).

**Online Figure II. Effects of inhibiting β_1_AR signaling by deletion of β_1_AR or inhibiting OCT3**. Cardiac function was recorded for 2 minutes at baseline and for 8 minutes after drug stimulation. The maximal cardiac response was reported. (**A, B**) β_1_AR-KO mice were injected with EPI (100 µg/kg, *i.p.*) or MAOi (0.4 mg/kg, 5 minutes). The maximal cardiac EF and HR was plotted. n = 8. (**C, D**) Inhibition of OCT3 by D-22 suppressed cardiac response to EPI in WT mice. WT mice were *i.p.* injected with EPI (100 µg/kg) in the presence or absence of pretreatment with D-22 (100 µg/kg, *i.p.*, 5 minutes). The maximal cardiac EF and HR was plotted. n = 9. Data are shown as mean ± SD. Two-tailed paired t-test was performed in panels (A, B). RM One-Way ANOVA was performed for panels (C, D) and followed by Tukey’s multiple comparison correction.

**Online Figure III. Effects of MAO-A inhibition on Ca^2+^ transient.** WT AVMs were loaded with 1 µmol/L Fluor-4 and paced at 1Hz. Traces show typical Ca^2+^ transient before (Basal) and after norepinephrine (NE), epinephrine (EPI), or dobutamine (DOB) stimulation in the absence or presence of pretreatment MAOi (5 µmol/L,5-minutes). NE = 0.1 µmol/L, EPI = 1 µmol/L, and DOB = 1 µmol/L.

**Online Figure IV. MAO-A inhibition enhances catecholamine-triggered β_1_AR signaling at the sarcoplasmic reticulum (SR).** WT AVMs expressing SR-AKAR3 biosensor were pretreated with MAOi, ICI and CGP for 5 minutes following by EPI stimulation. YFP/CFP FRET ratio was recorded for total 400s. **A**) Representative time courses and quantification of SR-PKA FRET responses upon EPI stimulation as indicated. **B**) The maximal FRET response after drug stimulation was plotted. Dot plots represent the mean ± SD of the indicated number of isolated AVMs from 3 rabbits. P values were obtained by One-way ANOVA followed by Tukey’s multiple comparison correction. EPI = 1 µmol/L, MAOi = 10 µmol/L, ICI 118,551 = 1 µmol/L and CGP 20,712a = 0.3 µmol/L.

**Online Figure V. MAO-A inhibition does not affect the PKA activity response to non-substrate DOB.** AVMs were treated with drugs as indicated for western blot. (**A**) The schematic depicts that MAOi does not degrade DOB and change the DOB-induced activation of β_1_AR. (**B, C**) Detection and quantification of phosphorylated PLB (pS16) and LTCC (pS1928) respond to DOB (1 µmol/L, 5 minutes) with or without pretreatment with MAOi (5 µmol/L). n = 3. (**D, E**) Detection and quantification of phosphorylated PLB (pS16) and LTCC (pS1928) respond to DOB (1 µmol/L, 5 minutes) with or without pretreatment with MAOi (5 µmol/L) and CORTI (2 µmol/L). n = 6. Dot plots represent mean ± SD. A.U.= arbitrary unit. P values were obtained by non-parametric analysis, the Kruskal-Wallis test with Dunn’s multiple comparison correction (**E**), or One-way ANOVA followed with Tukey’s multiple comparison correction (**C**).

**Online Figure VI. Generation and characterization of MAO-A cardiac-specific knockout (CKO) mouse.** (**A**) Generation of MAO-A-CKO (MHC-CRE, MAO-A ^flox/flox^) and MAO-A-FF (MAO-A ^flox/flox^) by crossbreeding MAO-A ^flox/flox^ with MHC-CRE mouse. (**B**) Genotyping of MAO-A-FF and CKO mice by PCR. (**C, D**) Co-staining of MAO-A (red color) and phospholamban (PLB, green color) in MAO-A FF and CKO AVMs. Pearson’s Coefficient quantification between MAO-A and PLB. Dot plots represent the mean ± SD of the indicated number of AVMs from 3 FF and 3 CKO mice. M-mode echocardiography was recorded for 2 minutes at baselines and for 8 minutes after drug administration. The maximal cardiac response was reported. (**E, F**) Quantification of EF before and after EPI or DOB stimulation (100 µg/kg, *i.p.*). n = 9. (**G**) Quantification of ΔEF = ∆EF_EPI_ (EF_EPI_ -EF_baseline_) or ∆EF_DOB_ (EF_DOB_ -EF_baseline_) in FF and CKO mice. n = 9. Data were shown as mean ± SD. P values were obtained by a two-tailed, unpaired Student’s *t*-test (panel **D, G**) or by a two-tailed, paired Student’s *t*-test (panel **E**, **F**).

**Online Figure VII. MAO-A deletion promotes local PKA activity in response to catecholamine.** MAO-A FF and CKO AVMs expressing AKAR3 biosensors were treated with EPI and DOB (1 µmol/L). YFP/CFP FRET ratio was recorded at the baseline and after drug stimulation. The maximal FRET responses were reported. **(A, C)** Agonist-response curves in SR-AKAR3 FRET ratio after stimulation with EPI (EC50, FF 793 ± 18 nmol/L, CKO 59 ± 2 nmol/L) or DOB (EC50, FF 134 ± 15 nmol/L, CKO 131 ± 14 nmol/L) in AVMs. Data represent mean ± SD of AVMs from 5 MAO-A-FF and 7 CKO mice. **(B, D)** Agonist-response curve of PM-AKAR3 FRET ratio after application of EPI (EC50, FF 228 ± 28 nmol/L; CKO 726 ± 29 nmol/L) or DOB (EC50, FF 240 ± 12 nmol/L, CKO 310 ± 15 nmol/L) in AVMs. Data represent the mean ± SD of AVMs from 5 MAO-A-FF and 7 CKO mice. P values were obtained by Two-way ANOVA with Tukey’s test.

**Online Figure VIII. Overexpression of MAO-A impairs catecholamine-induced intracellular β_1_AR signaling in rabbit AVMs.** Rabbit AVMs express AKAR3 biosensors together with MAO-A were stimulated with EPI, DOB, or NE as indicated. YFP/CFP FRET ratio was recorded at the baseline and after drug stimulation. The maximal FRET responses were reported. **(A, B)** Agonist dose-response curves of increases in PM-PKA FRET ratio are plotted (EC50 for EPI, OE-MAO-A 33.5 ± 5.3 nmol/L, Ctrl 9.2 ± 0.6 nmol/L; EC50 for NE, OE-MAO-A 6.1 ± 0.6 mmol/L, Ctrl 9.9 ± 0.7 nmol/L; EC50 for DOB, OE-MAO-A 114 ± 51 nmol/L, Ctrl 172 ± 55 nmol/L) and SR-PKA (EC50 for EPI, OE-MAO-A 3650 ± 257 nmol/L, CTRL 25.8 ± 2.5 nmol/L; EC50 for NE, OE-MAO-A 16.7 ± 4.0 mmol/L, Ctrl 12.3 ± 3.1 nmol/L; EC50 for DOB, OE-MAO-A 368 ± 32 nmol/L, Ctrl 181 ± 29 nmol/L). Data represent the mean ± SD of AVMs from 5 rabbits. **(C-E)** Rabbit AVMs (with or without MAO-A overexpression) were stimulated with EPI, DOB, or NE as indicated for 5 minutes. Western blots and quantification show the phosphorylation of PLB (pS16) and LTCC (pS1928). Data represent the mean ± SD. n = 5. P values were calculated by Two-way ANOVA with Tukey’s multiple comparison test.

**Online Figure IX. Interaction of MAO-A and OCT3 in MI mice.** (**A**) Representative Sirius red images of Ctrl and MI hearts from WT mice. (**B**) Representative echo images before and 3-days after MI. (**C**) Quantification of WT mice cardiac function before and 3-days after MI. n = 12. (**D, E**) Protein expression of β_1_AR, OCT3, and MAO-A in Ctrl and MI heart tissues. n = 4. (**F**) The cardiac effect of MAO-A and OCT3 inhibition in MI mice. MI mice were treated (*i.p.*) with MAOi (0.4 mg/kg, *i.p*.), CORTI (200 µg/kg, *i.p*.), or cotreated with MAOi+CORTI. M-mode echocardiography were recorded for 2 minutes at baseline and for 8 minutes post-drug stimulation. The maximal cardiac response was reported. n = 12. Data were shown as mean ± SD. P values were obtained by two-tailed, paired *t*-test (C), non-parametric Mann Whitney test (E), or RM One-way ANOVA analysis with Tukey’s multiple comparison correction (F).
